# Metastable Iron Sulfides Gram‐Dependently Counteract Resistant *Gardnerella Vaginalis* for Bacterial Vaginosis Treatment

**DOI:** 10.1002/advs.202104341

**Published:** 2022-02-05

**Authors:** Ling Fang, Ruonan Ma, Xuejiao J. Gao, Lei Chen, Yuan Liu, Yanwu Huo, Taotao Wei, Xiaonan Wang, Qian Wang, Haojue Wang, Chengjun Cui, Qifeng Shi, Jing Jiang, Lizeng Gao

**Affiliations:** ^1^ CAS Engineering Laboratory for Nanozyme Institute of Biophysics Chinese Academy of Sciences Beijing 100101 China; ^2^ Institute of Translational Medicine Department of Pharmacology School of Medicine Yangzhou University Yangzhou Jiangsu 225009 China; ^3^ Xishan People's Hospital of Wuxi City Wuxi Jiangsu 214105 China; ^4^ College of Chemistry and Chemical Engineering Jiangxi Normal University Nanchang Jiangxi 330022 China; ^5^ Joint Laboratory of Nanozymes in Zhengzhou University Academy of Medical Sciences Zhengzhou University Zhengzhou Henan 450052 China; ^6^ National Laboratory of Biomacromolecules Institute of Biophysics Chinese Academy of Sciences Beijing 100101 China

**Keywords:** bacterial vaginosis, Gram‐dependent, metastable iron sulfide, polysulfide species, resistant *Gardnerella vaginalis*

## Abstract

Bacterial vaginosis (BV) is the most common vaginal infection found in women in the world. Due to increasing drug‐resistance of virulent pathogen such as *Gardnerella vaginalis* (*G. vaginalis*), more than half of BV patients suffer recurrence after antibotics treatment. Here, metastable iron sulfides (mFeS) act in a Gram‐dependent manner to kill bacteria, with the ability to counteract resistant *G. vaginalis* for BV treatment. With screening of iron sulfide minerals, metastable Fe_3_S_4_ shows suppressive effect on bacterial growth with an order: Gram‐variable *G. vaginalis* >Gram‐negative bacteria>> Gram‐positive bacteria. Further studies on mechanism of action (MoA) discover that the polysulfide species released from Fe_3_S_4_ selectively permeate bacteria with thin wall and subsequently interrupt energy metabolism by inhibiting glucokinase in glycolysis, and is further synergized by simultaneously released ferrous iron that induces bactericidal damage. Such multiple MoAs enable Fe_3_S_4_ to counteract *G. vaginalis* strains with metronidazole‐resistance and persisters in biofilm or intracellular vacuole, without developing new drug resistance and killing probiotic bacteria. The Fe_3_S_4_ regimens successfully ameliorate BV with resistant *G. vaginalis* in mouse models and eliminate pathogens from patients suffering BV. Collectively, mFeS represent an antibacterial alternative with distinct MoA able to treat challenged BV and improve women health.

## Graphical abstract

Metastable iron sulfides (mFeS) demonstrate a Gram‐dependent antibacterial activity by releasing polysulfide species that penetrate bacteria with thin wall. The polysulfides then disrupt glycolytic energy metabolism and synergize with iron to kill bacteria without inducing new drug resistance. These mechanisms of action enable mFeS to counteract metronidazole‐resistant *Gardnerella vaginalis*, biofilm, and intracellular persister in bacterial vaginosis treatment.

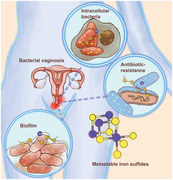

## 1 Introduction

Bacterial vaginosis (BV) is the most common vaginal infection among women with the population prevalence up to 29% in the world.^[^ ^1^ ^]^ The estimated economic burden for BV treatment may cost the world around $5 billion annually. Of clinical consequence, BV is associated with preterm birth and increased risk for acquisition of human immunodeficiency virus (HIV) and other sexually transmitted infections (STIs).^[^ ^2^ ^]^ Normally BV is treated with oral metronidazole, vaginal metronidazole gel, or vaginal clindamycin.^[^ ^3^ ^]^ Despite the availability of these treatments, more than half of women suffer recurrence of BV within in one year of treatment. As a primary pathogen in BV, *G. vaginalis* reside in vaginal flora which is normally predominated by the *Lactobacilli* species. But when *Gardnerella* bacteria become the dominant species, this leads to BV progress with the common symptom of vaginal discharge. *G. vaginosis* has been known to easily evolve metronidazole resistance, form persistent strains in biofilm or in host cells that protect them from antibiotics treatment.^[^ ^4^ ^]^ These resistance and persistence of pathogens result in the recurrence and relapse of BV. To tackle these issues, new concepts and approaches that different from traditional antibiotics mechanism are required for the discovery of antibacterial alternatives that can overcome bacterial resistance and persistence without developing new drug‐resistance and impacting probiotic species.^[^ ^5^ ^]^

The development of nanotechnology provides lots of cutting‐edged nanomaterials and strategies with nonantibiotic mechanisms against antimicrobial resistance.^[^ ^5^, ^6^ ^]^ For instance, silver nanoparticles demonstrate a strong antibacterial activity to resistant bacteria and biofilm by penetrating or release silver ion into bacterial microenvironment.^[^ ^7^ ^]^ Although highly effective, silver nanomaterials perform high toxicity to host cells and also cause bacterial resistance.^[^ ^8^ ^]^ Recently, a class of nanomaterials with enzyme‐like properties, which is termed as nanozymes, have shown promising capability to eliminate biofilm or intracellular bacteria.^[^ ^9^ ^]^ For instance, iron oxide nanozymes with peroxidase‐like activity can disrupt biofilm by degrading matrix and suppress intracellular bacteria.^[^ ^10^ ^]^ However, these nanozymes often rely on the presence of hydrogen peroxide (H_2_O_2_) to achieve bacterial killing, which reduces the biocompatibility and thus limits their application for in vivo antibacterial therapy. Our recent studies discovered that iron sulfide nanomaterials (nFeS) exhibited high antibacterial activity by sustained releasing iron and polysulfides. Impressively, the nFeS not only exhibited a broad spectrum to both Gram‐positive and Gram‐negative bacteria,^[^ ^11^ ^]^ but also eliminated biofilm and intracellular bacteria.^[^ ^12^ ^]^ Further studies revealed that the antibacterial activity was dependent on a ferroptosis‐like death in bacteria dominated by the released ferrous iron from nFeS under the condition without molecules that can chelate iron or resist oxidative damage.^[^ ^13^ ^]^ Unfortunately, the antibacterial performance of nFeS dramatically decreased for bacteria like *Escherichia*  *coli* or *Staphylococcus*  *aureus* in nutrient‐rich media or physiological environment, making them equivocal for in vivo antibacterial therapy.

**Figure 1:**
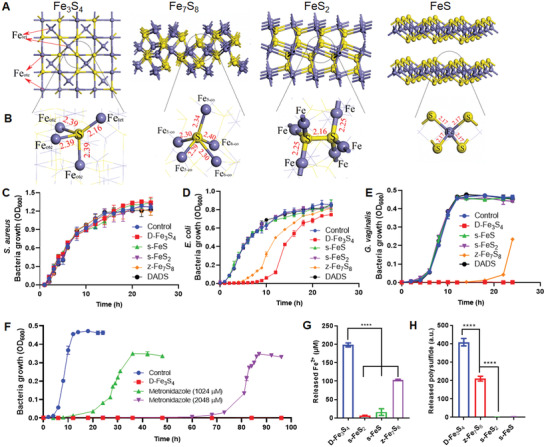
Metastable iron sulfides exhibit antibacterial activity in a Gram‐dependent manner. A,B) A schematic atom structure represents crystal phases of four iron sulfides with different bond length B) of Fe–S. Left to right: Fe_3_S_4_, Fe_7_S_8_, FeS_2_, FeS. C) Antibacterial activity of iron sulfides against Gram‐positive *S. aureus* in culture media. D) Antibacterial activity of iron sulfides against Gram‐negative *E. coli* in culture media. Both D‐Fe_3_S_4_ and z‐Fe_7_S_8_ exhibited partial inhibitory effects on *E. coli*. E) Antibacterial activity of iron sulfides against Gram‐variable *G. vaginalis* in culture media. D‐Fe_3_S_4_ exhibited the robust antibacterial activity followed by z‐Fe_7_S_8_. In contrast, neither s‐FeS nor s‐FeS_2_ exhibited any antibacterial effects. Concentration for z‐Fe_7_S_8_, s‐FeS and s‐FeS_2_ was adjusted to 500 × 10^‐6^ m. F) D‐Fe_3_S_4_ completely suppressed *G. vaginalis* without regrowth. In contrast, metronidazole (at 1024 × 10^‐6^ m or 2048 × 10^‐6^ m) cannot achieve complete suppression, as bacteria regrow after 24 h or 72 h. G) Fe^2+^ released from different iron sulfides. H) Polysulfides released from different iron sulfides. *n* = 3, ****p < 0.0001. Representative images are shown. Mean±SD are shown.

Here, we conducted a screen of iron sulfides including Fe_3_S_4_ (greigite), Fe_7_S_8_ (pyrrhotite), FeS and FeS_2_ (pyrite)^[^ ^14^, ^15^ ^]^ (**Figure** **1**A) and investigated their MoA against Gram‐positive *S. aureus*, Gram‐negative *E. coli* and Gram‐variable *Gardnerella vaginalis* under culture media conditions. Our experimental design allows to identify the exact crystal phase and components of iron sulfides in antibacterial process, as there are many crystal phases for iron sulfides and mutually transformation may occur between them. We found that metastable iron sulfides (mFeS), in particular for Fe_3_S_4_, exhibited highly antibacterial activity preferably against *G. vaginalis* through the MoAs of glycolysis inhibition and ferroptotic damage. This synergistic manner of action ensured that Fe_3_S_4_ counteracted the drug‐resistance of *G. vaginalis*, and demonstrated superior effects than silver nanoparticles and the first line medication of metronidazole for treating BV in mouse models and eliminating pathogens from clinical samples.

## 2 Results

### 2.1 Metastable Iron Sulfides Exhibit Antibacterial Activity with Gram‐Dependent Activity

To assess the activity of iron sulfides against bacteria under culture conditions, we first chose four types of iron sulfide phases both via nanosynthesis and from natural mineral sources, including DADS‐derived Fe_3_S_4_ (D‐Fe_3_S_4_), Zirantong (z‐Fe_7_S_8_, which was processed by a hyperthermia‐vinegar quenching method from traditional Chinese medicine), commercialized FeS (Sigma, s‐FeS), commercialized natural FeS_2_ (Sigma, s‐FeS_2_) respectively. The D‐Fe_3_S_4_ was synthesized using previous hydrothermal reaction with amended parameters to produce pure‐phase greigite.^[^ ^11^ ^]^ We then examined these minerals using SEM imaging. As shown in Figure S1A (Supporting Information), D‐Fe_3_S_4_ exhibited nanoscale morphologies of nanosheets and z‐Fe_7_S_8_ exhibited bulk block with nanorod‐like surface. In contrast, both s‐FeS and s‐FeS_2_ displayed as bulk blocks with flatten surface. Our subsequent X‐ray diffraction (XRD) analysis confirmed that the prepared D‐Fe_3_S_4_, z‐Fe_7_S_8,_ s‐FeS_2_ and s‐FeS were in pure phases of Fe_3_S_4_, Fe_7_S_8,_ FeS_2_ and FeS, respectively (Figure S1B, Supporting Information). A schematic of atom structures representing the phases of Fe_3_S_4_, Fe_7_S_8,_ FeS_2_ and FeS was shown according to the reported crystal information of iron sulfides (Figure 1A,B).

To test the antibacterial activity of D‐Fe_3_S_4_, z‐Fe_7_S_8,_ s‐FeS_2,_ and s‐FeS, we assessed the bacterial growth by measuring the optical density of the culture at 600 nm (OD_600_) in liquid media post 24 h. We first tested the inhibition efficiency of iron sulfides against Gram‐positive strain *S. aureus* as well as Gram‐negative strain *E. coli*. Surprisingly, we failed to detect any inhibitory effects for *S. aureus* using the above iron sulfides (at 500 × 10^‐6^ m) (Figure 1C), while we found that D‐Fe_3_S_4_ and z‐Fe_7_S_8_ exhibited partial inhibition at the early stages of growth for *E. coli* (Figure 1D).

We next tested *G. vaginalis* (GV14018) as a representative of Gram‐variable bacteria as it has a thin wall consisted of peptidoglycan without outer membrane, but appears to be Gram‐negative species under Gram staining.^[^ ^16^ ^]^ As shown in Figure 1E, *G. vaginalis* was completely suppressed in the presence of synthesized D‐Fe_3_S_4_ at 128 × 10^‐6^ m, and partially inhibited in the presence of z‐Fe_7_S_8_ at 500 × 10^‐6^ m. In contrast, neither s‐FeS nor s‐FeS_2_ inhibited bacterial growth. In addition, we determined the minimal inhibitory concentration (MIC) of D‐Fe_3_S_4_ against *G. vaginalis* and found that D‐Fe_3_S_4_ exhibited MIC at 89.6 × 10^‐6^ m, while metronidazole at 256 × 10^‐6^ m inhibited bacterial growth (Figure S1C,D, Supporting Information). When measuring bacterial availability using agar plate counting, we found that 99.99% of *G. vaginalis* bacteria were dead after 6 h incubation (Figure S1E, Supporting Information). Furthermore, while D‐Fe_3_S_4_ showed long‐term (up to 96 h) suppression of *G. vaginalis* growth at 128 × 10^‐6^ m, metronidazole failed to completely inhibit bacterial growth after 24 h (at 1024 × 10^‐6^ m) as well as 72 h (at 2048 × 10^‐6^ m) (Figure 1F). We also tested the antibacterial activity of DADS, an antibacterial ingredient in garlic oil that is used as a S donor for D‐Fe_3_S_4_ preparation. As shown in Figure 1C–E, DADS failed to inhibit the growth of *S. aureus*, *E. coli* or *G. vaginalis*. Together, these results showed that antibacterial activity of iron sulfides follows the order as: D‐Fe_3_S_4_> z‐Fe_7_S_8_>> s‐FeS and s‐FeS_2_. Among them, D‐Fe_3_S_4_ possesses the highest antibacterial activity and preferentially affects bacteria in the following order: Gram‐variable *G. vaginalis*>Gram‐negative *E. coli*>Gram‐positive *S. aureus*, indicating strict Gram‐selectivity. For all subsequent mechanisms and characterizations of antibacterial activity, we chose D‐Fe_3_S_4_. Further characterizations with high‐resolution SEM and TEM demonstrated that the lateral size and the thickness of D‐Fe_3_S_4_ nanosheets were at 491.10 ± 264.50 nm and 47.92 ± 10.72 nm (mean ± SD), respectively. The HR‐TEM patterns illustrated that the lattice fringe spacing of 0.298 nm was consistent with the interplanar distance of the (311) plane of the Fe_3_S_4_ phase and the electron diffraction pattern (the inset) indicated that the nanostructure was single crystal (Figure S2A–C, Supporting Information).

To better understand the differences in antibacterial activity of our four selected iron sulfides, we analyzed their ability to release iron and polysulfide into aqueous media. In our previous studies, we previously found that ferrous iron and polysulfides released from nanoiron sulfide are critical for antibacterial reactivity.^[^ ^11^, ^13^ ^]^ We thus speculated that the difference in antibacterial activity of D‐Fe_3_S_4_, z‐Fe_7_S_8_, s‐FeS and s‐FeS_2_ may derive from the efficiency of releasing ferrous iron and polysulfides. To confirm this hypothesis, we first analyzed the bond length of Fe–S in iron sulfide structure. In the spinal structure of Fe_3_S_4_,^[^ ^17^ ^]^ the length of 3 Fe_otc_–S bond is 2.39 Å (Figure 1B). Fe_7_S_8_ have three Fe_5‐co_–S bonds which are at 2.29–2.34 Å and two Fe_6‐co_–S bonds which are 2.30 and 2.40 Å,^[^ ^18^ ^]^ respectively (Figure 1B). In contrast, the Fe–S bonds in FeS and FeS_2_ are 2.17 Å^[^ ^19^ ^]^ and 2.25 Å,^[^ ^20^ ^]^ respectively (Figure 1B). Together, these data indicated that the Fe–S in Fe_3_S_4_ and Fe_7_S_8_ is easily broken upon iron and sulfur release. To test this hypothesis, we next measured that the release of ferrous iron and polysulfides with Iron Assay Kit (SigmaAldrich) and sulfane sulfur probe 4 (SSP4), respectively. We found that among the four iron sulfides, D‐Fe_3_S_4_ released ferrous iron most efficiently and z‐Fe_7_S_8_ showed half part of releasing, while FeS and s‐FeS_2_ showed negligible release of ferrous iron (Figure 1G). The release of polysulfides showed a trend similar to that of ferrous iron (Figure 1H). To identify the species of polysulfides, we used electrospray ionization mass spectrometry (ESI‐MS) and identified that persulfide (S_2_^2–^) and trisulfide (S_3_^2–^) were released (Figure S1F, Supporting Information). In addition, SEM images demonstrated that a structure transformation occurred as small curl structure formed on the surface of nanosheet of D‐Fe_3_S_4_ when dissolved in water for 72 h (Figure S2D,E, Supporting Information). Furthermore, energy dispersive X‐ray spectrometry (EDS) analysis of the sample of D‐ Fe_3_S_4_ precipitate (incubated in water for 72 h) showed a decreased atom ratio (atom%) of S (from 37.36% (0h) to 9.20% (72 h), while an increase ratio for oxygen (O) (Figure S2F–H, Supporting Information). Consistently, XRD analysis of D‐ Fe_3_S_4_ precipitate (incubated in water for 72 h) showed low and hetero peaks, which indicated that the precipitate was not the singular Fe_3_S_4_ with good crystallinity, but a mixture with poor crystallinity probably containing oxyiron compounds (Fe_3_O_4_ or Fe_2_O_3_) (Figure S2I, Supporting Information). These data demonstrated a transformation from iron sulfide to iron oxide during polysulfide species release in aqueous condition, further indicating the metastable property of D‐Fe_3_S_4_ under aqueous condition. Therefore, we hypothesized that the difference of releasing iron and polysulfide species is correlated with the antibacterial performance of the metastable iron sulfides.

### 2.2 Polysulfide Species Induce Gram‐Dependent Antibacterial Action

**Figure 2:**
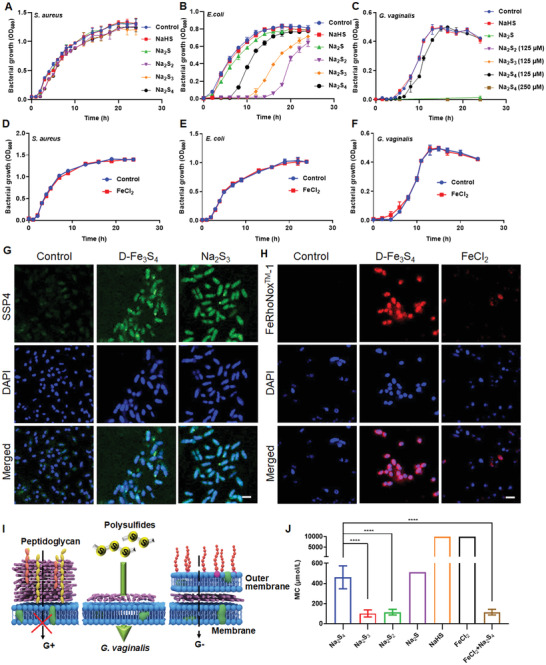
Polysulfide species exhibit Gram‐dependent antibacterial activity. A–C) Polysulfide species exhibited different antibacterial activity against bacteria. Polysulfide species (Na_2_S_2_, Na_2_S_3_ and Na_2_S_4_) failed to suppress the growth of A) Gram‐positive *S. aureus*, but partially suppress B) Gram‐negative *E. coli* , and heavily blocked growth of C) *G. vaginalis*. The concentration of polysulfide species was 500 × 10^‐6^ m if not specified. The significant suppression on *G. vaginalis* was achieved with Na_2_S_2_ (125 × 10^‐6^ m), Na_2_S_3_ (125 × 10^‐6^ m), and Na_2_S_4_ (250 × 10^‐6^ m), respectively. D–F) Iron (at 500 × 10^‐6^ m) failed to affect the growth of either D) *S. aureus*, E) *E. coli*, or F) *G. vaginalis*. G) Confocal imaging with SSP4 probe in *G. vaginalis* treated by D‐Fe_3_S_4_ or Na_2_S_3_. Scale bars: 1 µm. H) Confocal imaging with FeRhoNoxTM‐1 probe in *G. vaginalis* treated by D‐Fe_3_S_4_ or FeCl_2_. Scale bars: 10 µm. I) A schematic for antibacterial selectivity of polysulfide as a function of the bacterial wall. G+: Gram‐positive. G‐: Gram‐negative. J) Minimal inhibitory concentration (MIC) of polysulfide species for *G. vaginalis*. Both S_2_^2–^ and S_3_^2–^ showed high antibacterial activity, while S_4_^2–^ exhibited limited antibacterial effects by itself, though its effects were enhanced in the presence of iron. *n* = 3, ****p < 0.0001. Representative images are shown. Mean±SD are shown.

To confirm this hypothesis, we tested the antibacterial activity of both ferrous iron and polysulfide species in in vitro bacterial culture. In our previous study, we showed that under aqueous condition (e. g. water), ferrous iron induces ferroptotic damage in *S. aureus* and *E. coli*, which is synergized by glutathione (GSH) depletion mediated by polysulfide species.^[^ ^13^ ^]^ However, we found here that such antibacterial action is suppressed in culture media. In agreement with our earlier results, the D‐Fe_3_S_4_ showed high antibacterial activity to both *S. aureus* and *E. coli* in water, while polysulfide species including S_2_^2–^ and S_3_^2–^ only showed negligible bactericidal effect (Figure S3A–C, Supporting Information), indicating that polysulfides lack the ability to kill bacteria in water. In contrast, when the test was conducted in culture media (e.g., LB), the polysulfide species showed no inhibitory effects on Gram‐positive *S. aureus* (**Figure** **2**A) and *Streptococcus mutans* (*S. mutans*) (Figure S3D, Supporting Information), however, they partially suppressed the growth of Gram‐negative *E. coli*, *Pseudomonas aeruginosa* (*P. aeruginosa*) and *Acinetobacter baumannii* (*A. baumannii*) (Figure 2B, and Figure S3E,F, Supporting Information) and completely suppressed the growth of Gram‐variable *G. vaginalis* (Figure 2C). In the test for *E. coli*, S_2_^2–^, S_3_^2–^ and S_4_^2–^ showed significant inhibition (at 500 × 10^‐6^ m) on bacterial growth in the early stages of growth. In contrast, in the test for *G. vaginalis*, bacterial growth was completely suppressed at much lower concentration (at 128 × 10^‐6^ m) of S_2_^2–^ and S_3_^2–^. Even S_4_^2–^ exhibited achieved similar antibacterial effect at 250 × 10^‐6^ m. However, neither SH^–^ nor S^2–^ showed inhibition on *S. aureus*, *E. coli*, or *G. vaginalis*. Furthermore, ferrous iron failed to affect bacterial growth when cultured in media (Figure 2E,F). Together, these data clearly demonstrated that polysulfide species are able to suppress bacterial growth in the presence of culture media. The antibacterial potency follows below orders: S_3_^2–^>S_2_^2–^>S_4_^2–^>S^2–^>HS^–^.

The bacteria tested in our experiments can be divided into three types: I) *S. aureus* and *S. mutans* are Gram‐positive strains possessing thick bacterial wall. II) *E. coli*, *P. aeruginosa* and *A. baumannii* are Gram‐negative strains whose cell wall is thin wall yet protected by additional outer membrane; III) *G. vaginalis* consists of cell wall without LPS but appears Gram‐negative under the microscope ^[^ ^21^ ^]^ (Figure S3G,H, Supporting Information). We further characterized the wall thickness of the three bacteria using TEM and found that the wall thickness for *S. aureus*, *E. coli* and *G. vaginalis* were approx. 40.5 nm, 17.3 nm and 14.6 nm, respectively (Figure S3I,J, Supporting Information). Therefore, we speculated that antibacterial effects of polysulfide species occur in a Gram‐dependent manner, and thus is likely to be related to the structure of the bacterial wall. To confirm this, we characterized the entry of polysulfide species and ferrous iron into bacteria using either a SSP4 probe (Green) or a FeRhoNoxTM‐1 (Red) probe for our confocal microscopy analysis. As shown in Figure 2G, bright green signal was observed in *G. vaginalis* treated with D‐Fe_3_S_4_ or Na_2_S_3_, which indicated that either polysulfide species or the released moieties enter bacteria. Similarly, strong red signal was present in *G. vaginalis* treated with D‐Fe_3_S_4_ or FeCl_2_, indicating that ferrous iron readily accessed the bacterial wall (Figure 2H). However, we detected negligible signals for SSP4 and FeRhoNoxTM‐1 probes in *E. coli* and *S. aureus* in the presence of D‐Fe_3_S_4_, Na_2_S_3_ or FeCl_2_, indicating that neither polysulfide species nor ferrous iron access the barrier of bacterial wall (Figure S4A–D, Supporting Information). To verify that it is necessary for polysulfide species to cross the bacterial wall to exhibit antibacterial action, we next digested bacterial walls of *S. aureus* and *E. coli* using lysozyme. As shown in Figure S4E,F (Supporting Information), both D‐Fe_3_S_4_ and Na_2_S_3_ exhibited significant inhibitory effects on *S. aureus* predigested with lysozyme. In addition, both D‐Fe_3_S_4_ as well as Na_2_S_3_ completely blocked the growth of *E. coli* pre‐digested with lysozyme (Figure S4G, Supporting Information). CFU counting assay showed that Na_2_S_3_ killed all pre‐digested *E. coli* within 3 h in culture media (Figure S4H, Supporting Information). These results strongly suggest that the Gram‐specific effects of polysulfide species on bacterial growth are directly related to the differences in bacterial wall structures (Figure 2I).

In order to assess the antibacterial efficiency of polysulfide species, we next measured their minimal inhibitory concentration (MIC) using *G. vaginalis* as model bacteria in culture media. As shown in Figure 2J, S_2_^2–^, S_3_^2–^ exhibited the highest antibacterial activity against *G. vaginalis*, with MIC values of 115.2 × 10^‐6^ m for S_2_^2‐^ and 102.4 × 10^‐6^ m for S_3_^2–^, followed by S_4_^2–^ with MIC value at 460.8 × 10^‐6^ m. Surprisingly, we found that the presence of iron dramatically reduced the MIC of polysulfide species, especially for S_4_^2–^, for which we obtained values of 115.2 × 10^‐6^ m for *G. vaginalis*. This phenomenon suggested that iron acts synergistically with polysulfides. To identify the mechanism behind this observation, we prepared a mixture of Na_2_S_4_ and either ferrous or ferric chloride. The resulting colloid solution primarily consists of nanoparticles of iron sulfide (Figure S4I,J, Supporting Information). When we measured the amount of polysulfide species using SSP4 probe that strongly reacts with S_3_^2–^, we found that the added ferrous iron increased the S_3_^2–^ signal of Na_2_S_4_ (Figure S4K,L, Supporting Information). In particular, ferrous chloride generated more S_3_^2–^ species compared to ferric chloride. Therefore, iron may synergize the antibacterial activity of S_4_^2–^ by converting S_4_^2‐^ into S_3_^2–^ as the latter has superior antibacterial activity. Since our D‐Fe_3_S_4_ released both ferrous iron and polysulfides and performed the lowest MIC value to *G. vaginalis*, this feature may ensure that large amount of S_3_^2–^ is generated in the antibacterial process. Therefore, we chose D‐Fe_3_S_4_ for all subsequent experiments with *G. vaginalis*.

### 2.3 Polysulfide Induces Glycolysis Inhibition and Is Synergized by Ferrous Iron in *G. Vaginalis*

**Figure 3:**
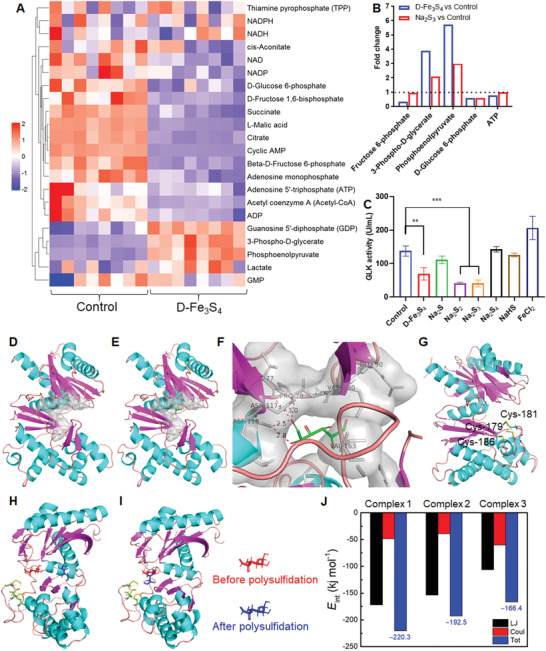
Polysulfide kills *G. vaginalis* by disrupting energy metabolism. A) The heatmap of metabolome analysis showing that abnormal change of intermediates occurred in the glycolytic pathway in *G. vaginalis*. A is control group and B is Fe_3_S_4_ treated group (*n* = 8). B) Incubation of *G. vaginalis* in D‐Fe_3_S_4_ and Na_2_S_3_ exhibited similar effects on glycolytic metabolism. C) Addition of D‐Fe_3_S_4_ inhibited glucokinase (GLK) activity in *G. vaginalis*. The polysulfide species, such as S_2_^2–^ and S_3_^2–^ also showed inhibitory effects on GLK. D) Predicted protein structure of glucokinase (*Gardnerella vaginalis* ATCC 14018 = JCM 11026, GenBank: BAQ33377.1) shows an active pocket. E) The final conformation of glucose and glucokinase complex after 20 ns MD simulation. F) The key residues around the glucose in the active center of glucokinase. G). Polysulfidation of cysteine residues (Cys‐179, Cys‐181, and Cys‐186) by polysulfides (S_3_^2–^). H) The binding conformations of glucose with glucokinase with the most negative interaction energy (*E*_int_) before (red, complex 1) and after (blue, complex 2) polysulfidation of cysteine residues. I) The binding conformations of glucose and glucokinase with similar ligand positions before (red) and after (blue, complex 3) polysulfidation of cysteine residues. J) The *E*_int_ between glucose and glucokinase in different binding conformations. *n* = 3, ***p < 0.001, ****p < 0.0001. Representative images are shown. Mean±SD are shown.

To assess how D‐Fe_3_S_4_ achieves selective antibacterial activity against *G. vaginalis*, we analyzed its effects at the biochemical level, energy metabolism level and transcription level. Since bacterial growth was suppressed in culture media, we therefore speculated that D‐Fe_3_S_4_ interferes with the metabolism of *G. vaginalis*. To confirm this hypothesis, we assessed the metabolome and transcriptome of *G. vaginalis* treated with D‐Fe_3_S_4_ under nonlethal conditions (50 × 10^‐6^ m for 1 h). As shown in **Figure** **3**A, the glycolytic metabolism of *G. vaginalis* was negatively influenced upon treatment with 50 × 10^‐6^ m D‐Fe_3_S_4_. Compared to untreated *G. vaginallis*, the levels of phosphoenolpyruvate (PEP) were increased 5.7‐fold in D‐Fe_3_S_4_‐treated bacteria compared to untreated *G. vaginalis*, while the levels of glucose 6‐phosphate and fructose 6‐phosphate decreased by 0.4‐fold and 0.68‐fold, respectively (Figure 3B). Next, we performed an in vitro enzyme assay to assess the effect of D‐Fe_3_S_4_ on glycolytic enzymes. Due to the decrease of Glucose 6‐phosphate, we thought the first step of glycolysis may be blocked. To this end, we cloned the gene of glucokinase in *G. vaginalis* and transformed the gene into *E. coli* for protein expression (Figure S5A, Supporting Information). For the purified glucokinase, we found that the activities of glucokinase (GLK), a key enzyme involved in glycolysis,^[^ ^22^ ^]^ was inhibited by D‐Fe_3_S_4_ (Figure 3C). The inhibition was also observed in the lysate of D‐Fe_3_S_4_‐treated bacteria (Figure S5B, Supporting Information). We next assessed the transcriptional levels of energy metabolism using a transcriptome assay. We found that starch, sucrose, glycolysis/gluconeogenesis pathways were downregulated and the transcription for PTS sugar transporter subunit (EIIC) was increased in the presence of D‐Fe_3_S_4_ (Figure S5C and Table S1, Supporting Information), which further confirms the lethal effects of D‐Fe_3_S_4_ on the energy metabolism of *G. vaginalis*. Accumulation of PEP and the lack of glucose 6‐phosphate are reliable indicators showing that the bacteria attempt to regulate bacterial phosphotransferase system (PTS) to acquire energy for bacteria growth.^[^ ^23^ ^]^ The levels of ABC transporters related to sugar import processes^[^ ^24^ ^]^ were also significantly suppressed (Figure S5C, Supporting Information). Together, these features clearly demonstrated that D‐Fe_3_S_4_ causes cell death of *G. vaginalis* by disrupting energy metabolism.

When we treated *G. vaginalis* with 50 × 10^‐6^ m Na_2_S_3_, we found that Na_2_S_3_ exerted similar effects on metabolic and transcriptome levels to those caused by D‐Fe_3_S_4_ (Figure S5D and Table S2, Supporting Information). Importantly, we found that GLK activity was also inhibited by polysulfides, thus showing that GLK activity is not affected by the presence of iron (Figure 3C). To understand the inhibitory mechanism of polysulfides, we predicted the 3D structure of GLK (*Gardnerella vaginalis* ATCC 14 018 = JCM 11 026, GenBank: BAQ33377.1) in *G. vaginalis* using I‐TASSER server. As shown in Figure 3D, the *G. vaginalis* GLK structure comprises a typical pocket‐like active center. We then predicted glucose binding to the pocket using molecular dynamic (MD) calculation, which showed that the glucose (green molecule) bound to the inner side of the pocket and interacted with residues including Pro78, Asn117, Asp118, Val163 (Figure 3E,F). In addition, it clearly showed that a proximal loop connecting two *β*‐sheets extended into the pocket. There are three cysteine residues located on the loop, which are Cys179, Cys181, and Cys186 (Figure 3G). We speculated that the thiols in these residues are the target of polysulfides to inhibit GLK activity. To confirm it, we first evaluated the feasibility of polysulfides (S_3_^2–^) reacting with thiol group in cysteine by calculating the energy barrier under aqueous condition (physiological pH, 37 ℃). It showed that the energy barrier for the reaction of S_3_^2–^ and thiol group was only 15.6 kcal mol^–1^, indicating the reaction readily occurs under physiological condition. This result suggested that S_3_^2–^ can react with the three cysteine residues on proximal loop of active pocket in GLK (Figure 3G). We then analyzed the binding conformation and calculated the values of interaction energy (*E*_int_) for glucose with GLK before and after polysulfidation. Before polysulfidation, the GLK combines with glucose in its active pocked (complex1, Figure 3E,F) with an *E*_int_ of −220.3 kJ mol^–1^ (Figure 3J). Notably, the best binding site of glucose and GLK changed after the polysulfidation of cysteine residues as denoted in Figure 3H where the glucose was shown in blue. The corresponding *E*_int_ was ‐192.5 kJ mol^–1^ (complex 2, Figure 3J). The relative position of glucose before the polysulfidation of GLK was illustrated in red (Figure 3H) as well for clarity. If the glucose was docked into polysulfidated GLK at the location similar to that of the complex 1, as illustrated in Figure 3I, their *E*_int_ increased to ‐166.4 kJ mol^–1^ (complex 3, Figure 3J), which indicates that the polysulfidation was adverse for glucose binding to the active center of GLK. Collectively, based on MD analyses, the inhibition of polysulfides on GLK activity is possibly through the polysulfidation of cysteine restudies proximally located on the active center of GLK.

**Figure 4:**
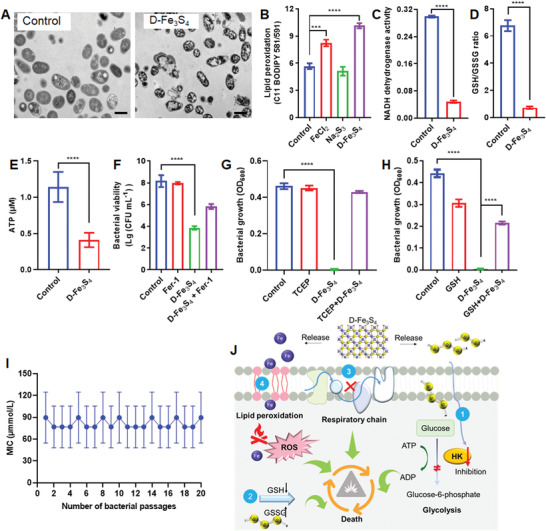
Iron synergizes antibacterial activity by inducing ferroptotic damage. A) TEM image of *G. vaginalis* treated with D‐Fe_3_S_4_ showing leakage of intracellular cytoplasm. Concentration for D‐Fe_3_S_4_ was 100 × 10^‐6^ m. Scale bar = 500 nm. B) Lipid peroxidation in *G. vaginalis* induced by D‐Fe_3_S_4_. FeCl_2_ alone resulted in high levels of lipid peroxidation, while Na_2_S_4_ failed to induce lipid peroxidation, indicating that the increase in lipid peroxidation was caused by iron. C) The respiratory chain of *G. vaginalis* was interrupted by D‐Fe_3_S_4_. The activity of NADH dehydrogenase in complex I of respiratory chain was dramatically reduced. D) The ratio of GSH/GSSG decreased upon D‐Fe_3_S_4_ treatment, indicating that D‐Fe_3_S_4_ is causing GSH depletion in *G. vaginalis*. E) ATP production was reduced in *G. vaginalis* upon D‐Fe_3_S_4_ treatment, indicating that energy metabolism was affected by D‐Fe_3_S_4_. F–H) the inhibition of D‐Fe_3_S_4_ on *G. vaginalis* is counteracted by F) Ferrostatin‐1 (inhibitor of ferroptosis), TCEP (an iron chelator and reducing agent of G) S–S bond), and H) GSH. I) Drug resistance of *G. vaginalis* treated under sub‐lethal concentration (32 × 10^‐6^ m) of D‐Fe_3_S_4_. The MIC remained constant after 20 passages of *G. vaginalis*. J) A schematic summary of the mechanisms causing bacterial death of *G. vaginalis* upon ferrosulfucide effect induced by iron polysulfide. 1, glucokinase inhibition, 2, glutathione (GSH) depletion, 3, respiratory chain depression, 4, lipid peroxidation. *n* = 3, ***p < 0.001, ****p < 0.0001. Representative images are shown. Mean±SD are shown.

In addition to the changes on metabolome and transcriptome, we also tested the ability of D‐Fe_3_S_4_ to damage the bacterial morphology using TEM imaging. As shown in **Figure** **4**A, when treated with 100 × 10^‐6^ m D‐Fe_3_S_4_, the bacterial cells exhibited a deformed surface structure. In addition, the cytosol displayed a large hollow area, indicating that the membrane was disrupted and the cytoplasm content leaked into the extracellular space. To assess lipid peroxidation in *G. vaginalis*, we measured the fluorescent intensity using a C11 BODIPY probe, which stains lipid ROS. As shown in Figure 4B, lipid peroxidation increased when *G. vaginalis* was treated with either D‐Fe_3_S_4_ or FeCl_2_. In contrast, we detected no signal for *G. vaginalis* treated with Na_2_S_3_. When we used an iodonitrotetrazolium chloride assay to assess respiration of *G. vaginalis*, we found that the respiratory chain of complex I (NADH dehydrogenase) was inhibited (Figure 4C). Furthermore, when we measured the level of glutathione in *G. vaginalis* treated with D‐Fe_3_S_4_, we found that the ratio of GSH/GSSG was considerably lower compared to levels in untreated bacteria (Figure 4D), indicating that GSH was depleted in *G. vaginalis* upon D‐Fe_3_S_4_ treatment. When we assessed the energy level of bacteria cells using an ATP kit, we found that ATP levels decreased upon D‐Fe_3_S_4_ treatment in *G. vaginalis* (Figure 4E), which confirmed that the energy metabolism was interfered by D‐Fe_3_S_4_.

Our observed changes in morphology, lipid peroxidation, GSH depletion and respiratory chain function indicated that treatment of *G. vaginalis* with D‐Fe_3_S_4_ caused ferroptotic damages, ultimately resulting in bacterial death. To confirm these findings, we performed gain of function experiments using inhibitors of ferroptosis. As shown in Figure 4F, addition of ferrostatin‐1, a ferroptosis inhibitor that scavenges lipid peroxide, reduced the antibacterial activity of D‐Fe_3_S_4_. When we added TCEP, an iron chelator that also reduces polysulfide, we found that TCEP strongly inhibited antibacterial activity of D‐Fe_3_S_4_ (Figure 4G). Importantly, addition of GSH also reduced the antibacterial activity of D‐Fe_3_S_4_ (Figure 4H). These data strongly suggested that D‐Fe_3_S_4_ primarily induces ferroptotic damages in *G. vaginalis* affected. Presumably, this ferroptotic damage is mainly the result of the iron present. When incubating bacteria with D‐Fe_3_S_4_ in water, both iron ions (in particular for Fe^2+^) and D‐Fe_3_S_4_ caused cell death in *G. vaginalis* as indicated by lipid peroxidation (Figure S5E,F, Supporting Information). Another chelating agent, EDTA, achieved the same effects as TCEP by chelating iron (Figure S5G, Supporting Information). However, when we incubated *G. vaginalis* with D‐Fe_3_S_4_ in culture media, D‐Fe_3_S_4_ inhibited cell growth of *G. vaginalis*, not ferrous or ferric iron (Figure S5H, Supporting Information), indicating that iron induced ferroptotic damages in the antibacterial action of D‐Fe_3_S_4_ represents an auxiliary effect.

The above dual antibacterial mechanisms target metabolism inhibition and oxidative damage in bacteria, which may prevent drug resistance. To prove this, we incubated *G. vaginalis* under sublethal concentration (32 × 10^‐6^ m) of D‐Fe_3_S_4_ and monitored changes of the MIC values. As shown in Figure 4I, MIC values remained constant after 20 passages of *G. vaginalis* under continuous pressure of D‐Fe_3_S_4_, indicating that bacteria did not generate drug resistance to D‐Fe_3_S_4_. Together, these data clearly demonstrated that the polysulfides rather than the iron are responsible for inhibiting the glycolytic energy metabolism in *G. vaginalis*, which is the primary factor causing bacterial death in culture media. Moreover, both D‐Fe_3_S_4_ and Na_2_S_3_ reduced the transcription of vaginolysin (VLY) in *G. vaginalis* which is a key virulent factor causing damage to vaginal epithelial cells^[^ ^25^ ^]^ (Figure S5I, Supporting Information). Taken together, these results reveal that Fe_3_S_4_ kills *G. vaginalis* by integrating polysulfide‐induced suppression on energy metabolism and iron‐induced ferroptotic damage, which is termed as ferrosulfucidal effect (Figure 4J).

### 2.4 Metastable Iron Sulfide Counteracts Antibiotic Resistance of *G. Vaginalis*

**Figure 5:**
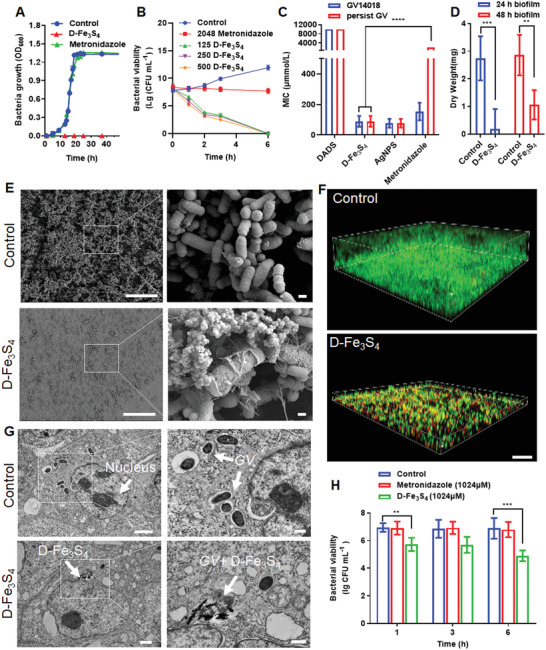
D‐Fe_3_S_4_ counteracts antibiotic resistance of *G. vaginalis*. A,B) D‐Fe_3_S_4_ suppressed metronidazole‐resistant strain of *G. vaginalis* (MRGV) by A) measuring bacterial growth (OD600) and B) viability (CFU mL^–1^). While metronidazole at 2048 × 10^‐6^ m showed no any inhibition on MRGV, D‐Fe_3_S_4_ at 125 × 10^‐6^ m, 250 × 10^‐6^ m or 500 × 10^‐6^ m killed GRSV eventually within 6 h (B). C) D‐Fe_3_S_4_ killed tolerant *G. vaginalis* induced by metronidazole. The tolerant bacteria were challenged with metronidazole stress and regrown in fresh culture media. D‐Fe_3_S_4_ achieved identical MIC for both tolerant and normal strain of *G. vaginalis*, while metronidazole failed to inhibit persistent strain. D–F) D‐Fe_3_S_4_ suppressed *G. vaginalis* biofilm characterized by D) dry weight assay, E) SEM imaging, and F) 3D imaging with confocal microscopy. Scale bars E): left side 50 µm; right side 300 nm. Scale bars F): 30 µm. G,H) D‐Fe_3_S_4_ suppressed intracellular *G. vaginalis* in VK2 cells characterized by G) TEM imaging and H) CFU counting. Scale bars: left side 2 µm, right side 500 nm. *n* = 3, **p < 0.01, ***p < 0.001, ****p < 0.0001. Representative images are shown. Mean±SD are shown.

*G. vaginalis* is characterized by resistance to antibiotic treatments, resulting in common failure of antibiotic treatment and ultimate recurrence of vaginosis. It remains unclear whether D‐Fe_3_S_4_ represents a suitable candidate for clinical treatment of *G. vaginalis*. To evaluate the clinical use of D‐Fe_3_S_4_, we first established four resistance models, namely antibiotics‐resistant bacteria, antibiotics‐induced persistent bacteria, biofilm‐embedded bacteria, as well as intracellularly growing bacteria. In the model of antibiotic‐resistant *G. vaginalis*, we exposed a clinical strain that was determined to be resistant to metronidazole (henceforth called MRGV) (Figure S6A, Supporting Information) to a serial dilution of D‐Fe_3_S_4_. We monitored cell growth with OD_600_ measurement of BHIs containing MRGV and D‐Fe_3_S_4_. As shown in **Figure** **5**A, MRGV was susceptible to D‐Fe_3_S_4_ growth inhibition, while metronidazole failed to inhibit bacterial growth. When we used a CFU counting assay, we found that 125 × 10^‐6^ m D‐Fe_3_S_4_ caused 100% cell death within 6 h, while 2048 × 10^‐6^ m metronidazole showed no inhibitory effects (Figure 5B). Next, we used metronidazole to challenge wild‐type *G. vaginalis* for 10 generations and collected the regrowing *G. vaginalis* as the tolerant strain. When we conducted MIC assay, we found that the tolerant *G. vaginalis* (challenged with 1024 × 10^‐6^ m metronidazole for 10 generations) showed the reduced susceptibility to metronidazole with a MIC up to 2048 × 10^‐6^ m (Figure 5C), compared to primary *G. vaginalis* whose MIC of metronidazole was 153.6 × 10^‐6^ m. Strikingly, D‐Fe_3_S_4_ showed same MIC at 89 × 10^‐6^ m to metronidazole persistent *G. vaginalis* and normal strain.

*G. vaginalis* also develops biofilm‐embedded or intracellular forms to escape antibiotic treatment.^[^ ^4^, ^26^ ^]^ *G. vaginalis* is able to form a biofilm on vaginal epithelium, which is thought to play an important role in drug resistance and relapsing infection.^[^ ^27^ ^]^ We next evaluated the antibacterial activity of D‐Fe_3_S_4_ against *G. vaginalis* residing in biofilms. To this end, we constructed a biofilm model by culturing *G. vaginalis* on the surface of a glass slide. As shown in Figure S6B (Supporting Information), mature *G. vaginalis* biofilm with typical bacterial colonies and biofilm matrix was formed after 24 h. Next, we analyzed the effect of D‐Fe_3_S_4_ treatment on mass, morphology and bacterial viability in mature biofilm using dry weight assay, crystal violet staining, SEM, confocal microscopy and CFU counting. As shown in Figure 5D, the dry weight of a 24 h biofilm prior to treatment was approximately 2.7 mg per biofilm. Upon D‐Fe_3_S_4_ treatment, the dry weight decreased to 0.18 mg per biofilm. Similarly, upon D‐Fe_3_S_4_ treatment, the dry weight of 48 h biofilm was reduced from 2.8 mg per biofilm to 1.06 mg per biofilm. The staining assay with crystal violet verified the similar change in mature biofilm upon D‐Fe_3_S_4_ treatment (Figure S6C,D, Supporting Information). In addition, if the 24 h biofilm was treated with D‐Fe_3_S_4_, it stopped growing in the following 24 h incubation (Figure S6E–G, Supporting Information). Further imaging assays with SEM and confocal microscopy confirmed that *G. vaginalis* biofilm was disrupted dramatically in the presence of D‐Fe_3_S_4_ (Figure 5E,F, Movies S1 and S2, Supporting Information). CFU counting showed that D‐Fe_3_S_4_ reduced more than 4‐log of bacteria number per biofilm (Figure S6G, Supporting Information). Importantly, the biofilm established with metronidazole resistant strain of *G. vaginalis* was also suppressed by D‐Fe_3_S_4_, while the biofilm established with either normal or metronidazole‐resistant *G. vaginalis* was not inhibited by metronidazole (Figure S6H,I, Supporting Information).

*G. vaginalis* also can be internalized into epithelium cell to evade antibiotic killing.^[^ ^26^ ^]^ To assess the cellular antibacterial activity of D‐Fe_3_S_4_, we then constructed an intracellular model of *G. vaginalis*. Human immortalized VK2 cells (ATCC 2616) were infected with *G. vaginalis*, and internalization of bacteria was characterized using both TEM as well as CFU counting assay. When we evaluated the morphology of intracellular *G. vaginalis* using TEM, we found that *G. vaginalis* was readily internalized into VK2 cells (6–8 bacteria per cell), but the bacteria were found to be deformed in the presence of D‐Fe_3_S_4_, which appeared to enter cytoplasm of VK2 (Figure 5G). When we evaluated the viability of intracellular *G. vaginalis* using CFU counting assay, we found that D‐Fe_3_S_4_ (1024 × 10^‐6^ m) reduced bacteria numbers by 2‐log10 in VK2 cells within 6 h. In comparison, metronidazole showed negligible antibacterial effects under identical conditions (Figure 5H). Together, these results clearly demonstrated that D‐Fe_3_S_4_ are able to kill *G. vaginalis* regardless of antibiotic‐resistance or persistence, eliminate *G. vaginalis* embedded in biofilm and suppress intracellular *G. vaginalis* hiding in epithelial cells, thus showing that D‐Fe_3_S_4_ treatment successfully counteracts the antibiotic‐resistance developed by *G. vaginalis*.

### 2.5 D‐Fe_3_S_4_ Is Biocompatible with Probiotic *Lactobacillus* and Nontoxic to Epithelial Cells

**Figure 6:**
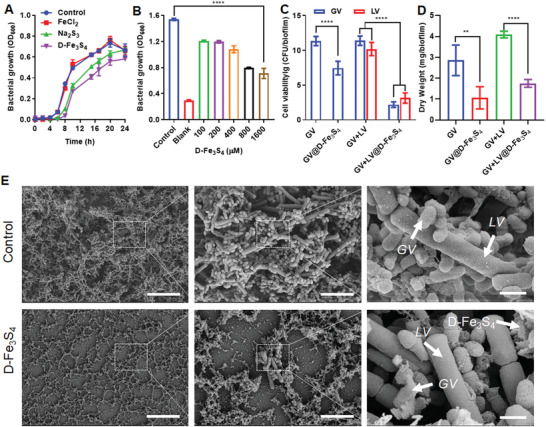
D‐Fe_3_S_4_ is biocompatible with probiotic *lactobacillus*. A,B) D‐Fe_3_S_4_ showed minimal antibacterial activity to vaginal lactobacillus in culture media with A) different growth time and B) concentration. D‐Fe_3_S_4_ or Na_2_S_3_ at 500 × 10^‐6^ m only partially suppressed the growth of A) *lactobacillus*, B) even D‐Fe_3_S_4_ at 1600 × 10^‐6^ m only suppressed 50% growth, indicating a high value of MIC (>1600 × 10^‐6^ m) for D‐Fe_3_S_4_ toward *lactobacillus*. C–E) D‐Fe_3_S_4_ suppressed mixed species biofilm composed of *G. vaginalis* and vaginal *lactobacillus* as assessed by C) CFU counting, D) dry weight assay, and E) SEM imaging. CFU counting showed that vaginal *lactobacillus* became prevalent in mixed‐species biofilm treated by D‐Fe_3_S_4_. SEM imaging showed that *G. vaginalis* was deformed while vaginal *lactobacillus* remained intact in the mixed‐species biofilm treated by D‐Fe_3_S_4_. Scare bars: left side 25 µm, middle 5 µm, right side 1 µm. GV: *G. vaginalis*, LV: *vaginal lactobacillus*. *n* = 3, **p < 0.01, ****p < 0.0001. Representative images are shown. Mean±SD are shown.

Vaginal *lactobacillus* (LV) is a Gram‐positive probiotic bacterium that exists in a symbiotic relationship with *G. vaginalis* under biofilm condition. LV forms an acid microenvironment and produces H_2_O_2_, thus controlling the balance between species present in the vaginal microbiota.^[^ ^28^ ^]^ To assess the biocompatibility of D‐Fe_3_S_4_, we evaluated the viability of vaginal lactobacillus following exposure to various concentrations of D‐Fe_3_S_4_. As shown in Figure 2J, *G. vaginalis* growth in the presence of D‐Fe_3_S_4_ is characterized by a MIC at 89.6 × 10^‐6^ m. In contrast, LV growth was largely unaffected, as indicated by D‐Fe_3_S_4_ up to 1600 × 10^‐6^ m (**Figure** **6**A,B). In agreement with these results, D‐Fe_3_S_4_ failed to affect cell viability of vaginal *lactobacillus* under in vitro aqueous condition (water), even after exposure for 3 h incubation (Figure S6J, Supporting Information). To compare the biosafety of D‐Fe_3_S_4_, silver nanoparticles (AgNPs), which are characterized by high antibacterial activity, were tested in parallel. Silver nanoparticles exhibited antibacterial activity against *G. vaginalis* with a MIC value of 76.8 × 10^‐6^ m. However, the silver particles also showed stronger suppression on vaginal *lactobacillus* with MIC value at 256 × 10^‐6^ m. We found that under in vitro aqueous conditions, 100 × 10^‐6^ m silver nanoparticles caused a four‐log reduction in vaginal l*actobacillus* cell numbers after only 1 h incubation (Figure S6K, Supporting Information). In addition, our cytotoxicity assay showed that silver nanoparticles at 64 × 10^‐6^ m caused cell death of more than 90% VK2 cells (Epithelial cell line from human normal vaginal mucosal tissue) (Figure S6L, Supporting Information). In contrast, D‐Fe_3_S_4_ only caused cell death of 11% VK2 cells, at concentration as high as 512 × 10^‐6^ m (Figure S6M, Supporting Information) and the released polysulfides and ferrous iron showed no any cytotoxicity to VK2, RAW264.7 (macrophages from mouse) and HaCaT (The immortalized human keratinocytes) (Figure S6N–P, Supporting Information).

To test if D‐Fe_3_S_4_ prefers to kill virulent pathogen, we prepared a dual species biofilm with *G. vaginalis* and vaginal *lactobacillus*. As shown in Figure 6C, exposure to D‐Fe_3_S_4_ reduced *G. vaginalis* growth by 9 logs. As a result, LV established itself as the dominant strain following D‐Fe_3_S_4_ treatment for 12 h. Next, we assessed dry weight of dual species biofilm exposed to D‐Fe_3_S_4_ treatment. As shown in Figure 6D, the influence on dry weight of biofilm mass was also reduced dramatically. SEM images showed that vaginal *lactobacillus* retained intact shape and all *G. vaginalis* were disrupted in the dual species biofilm (Figure 6E). Together, these results clearly demonstrated that D‐Fe_3_S_4_ is superior to silver nanoparticles in its biocompatibility with both probiotic bacteria and vaginal cells.

### 2.6 D‐Fe_3_S_4_ Is a Suitable Candidate for Treating Bacterial Vaginitis

**Figure 7:**
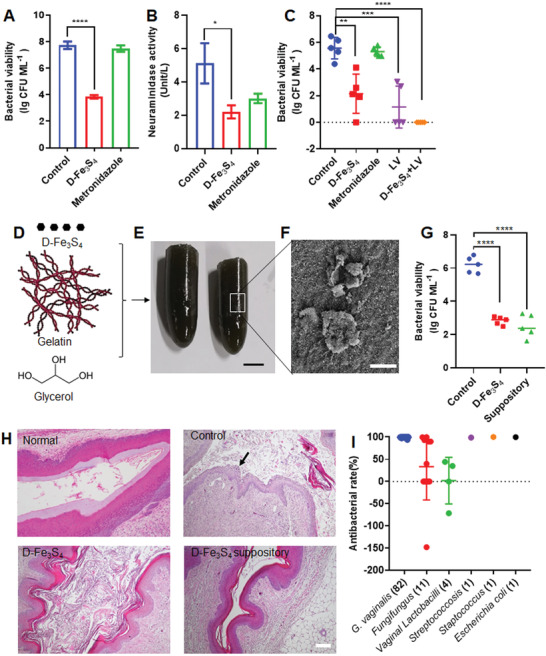
D‐Fe_3_S_4_ is able to treat bacterial vaginosis in mice and clinical samples. A) CFU counting showed D‐Fe_3_S_4_ reduced the number of *G. vaginalis* in mice, while metronidazole failed. B) Neuraminidase activity of *G. vaginalis* was reduced following treatment with D‐Fe_3_S_4_ and metronidazole, indicating that the virulence of *G. vaginalis* was reduced. C) Combination therapy of D‐Fe_3_S_4_ and probiotic *lactobacillus* in the model infected with metronidazole‐resistant *G. vaginalis*. Both D‐Fe_3_S_4_ alone and *lactobacillus* alone showed considerable suppression on bacterial growth compared to metronidazole, however, combination of D‐Fe_3_S_4_ and *lactobacillus* showed maximum therapeutic effects. D) A schematic of a suppository prepared from D‐Fe_3_S_4_, gelatin and glycerol. E) The prepared suppository containing 1 mg mL^–1^ D‐Fe_3_S_4_. Scale bar 1 cm. F) SEM image of sectioned suppository composed of D‐Fe_3_S_4_. Scale bar 2 µm. G) D‐Fe_3_S_4_ suppository showed comparable antibacterial effects in mouse model with D‐Fe_3_S_4_ treatment. H) Histochemistry staining demonstrated that the vaginal tissue recovered quickly in the group treated with D‐Fe_3_S_4_ and D‐Fe_3_S_4_ suppository compared to control group (the red arrow indicated a microcystis). The normal group represents the mouse without infection. Scale bar = 100 µm. I) Statistical analysis of 100 clinical samples from patients with bacterial vaginosis following the treatment with D‐Fe_3_S_4_. 82 samples were confirmed with *G. vaginalis* infection and could be eliminated completely. Three samples infected with *Streptococcosis*, *Staptococcus* or *E. coli* also can be eliminated, while 11 samples infected with Fungi and four samples with *lactobacillus* showed very limited or no antibacterial effect. *n* = 5, *p < 0.05, **p < 0.01, ***p < 0.001, ****p < 0.001. Representative images are shown. Mean±SD are shown.

Given that D‐Fe_3_S_4_ exhibited robust antibacterial activity against *G. vaginalis* and high biocompatibility to probiotics and vaginal cells, we sought to determine if D‐Fe_3_S_4_ can be used to treat bacterial vaginosis. We then evaluated the therapeutic effects of D‐Fe_3_S_4_ in a bacterial vaginitis model. To this end, we infected female Balb/C mice with *G. vaginalis*. Mice infected for 3 d (1 × 10^8^ CFU/mouse/day) were treated with D‐Fe_3_S_4_ by direct injection of 10 µL D‐Fe_3_S_4_ (500 × 10^‐6^ m) or 10 µL of metronidazole (500 × 10^‐6^ m) into the vagina once per day for 7 d. As shown in Figure S7A (Supporting Information), the infected mice discharged large amount of vaginal secretion. The group treated with D‐Fe_3_S_4_ showed negligible vaginal secretion 4 d postinfection, while those treated with metronidazole reached the similar effect 8 d postinfection. Ten days postinfection, the mice were sacrificed and the vaginal secretion was assessed for viability of *G. vaginalis* using histochemistry, CFU counting and enzyme assay. Our histochemistry analysis showed that the vaginal epithelial tissue of mice treated with D‐Fe_3_S_4_ recovered on 8 days post infection (Figure S7B, Supporting Information). We determined bacterial viability of in vaginal tissues using CFU counting. As shown in **Figure** **7**A, the group treated with D‐Fe_3_S_4_ exhibited the lowest number of colonies (3.83 lg (CFU mL^–1^)), while those without treatment or treated with metronidazole exhibited colonies number at 7.72 and 7.47 lg (CFU mL^–1^), respectively. Next, we used neuraminidase assay to determine the virulence of *G. vaginalis*. Our analysis showed that the activity of neuraminidase was reduced in the group treated with D‐Fe_3_S_4_ (Figure 7B), further indicating that the growth of vaginal bacteria was suppressed.^[^ ^29^ ^]^

To confirm the therapeutic effect of D‐Fe_3_S_4_, we used the metronidazole‐resistant strain MRGV to construct another in vivo infection model. To this end, we introduced a combination treatment consisting of D‐Fe_3_S_4_ and probiotic LV. LV had previously been recognized as a promising treatment for vaginosis.^[^ ^30^ ^]^ As shown in Figure S7C (Supporting Information), the mice infected with MRGV failed to recover from the infection by treatment with metronidazole alone. However, upon treatment with D‐Fe_3_S_4_, LV or D‐Fe_3_S_4_+LV, these mice recovered within 7 d. When we evaluated bacterial viability of *G. vaginalis* in vaginal tissues using CFU counting assay, we found that D‐Fe_3_S_4_ alone and LV alone reduced the number of *G. vaginalis* from 5.3 lg (CFU mL^–1^) to 2.1 and 1.1 lg (CFU mL^–1^) In contrast, a combination of D‐Fe_3_S_4_ and LV showed the best therapeutic effect, characterized by bacterial death of nearly 100% (Figure 7C). All treatments with D‐Fe_3_S_4_ as well as a combination of D‐Fe_3_S_4_ and LV showed little effect on the body weight of treated mice (Figure S7D, Supporting Information). Histochemistry analysis showed that the organs including heart, liver, spleen, lung, kidney and uterus are not affected after 7 d continuously vaginal administration, indicating good biocompatibility of D‐Fe_3_S_4_ with in vivo system (Figure S7E, Supporting Information).

To render D‐Fe_3_S_4_ suitable for in vivo application, we prepared a suppository by mixing D‐Fe_3_S_4_ with gelatin and glycerol (Figure 7D–F), with the characteristic of dissolution to release D‐Fe_3_S_4_ under physiological conditions (e.g., water, 37 ℃). We found that the suppository with 1 mg mL^–1^ D‐Fe_3_S_4_ was solid with black color (Figure 7E) and the inside D‐Fe_3_S_4_ remained sheet‐like structure as characterized by SEM (Figure 7F), indicating that D‐Fe_3_S_4_ is stable in this suppository. Using our CFU counting assay, we found that D‐Fe_3_S_4_ suppository (After one year storage at room temperature) exhibited MIC at 117 × 10^‐6^ m to both GV14018 and MRGV, indicating that its antibacterial activity is comparable with free D‐Fe_3_S_4_ and stable for long‐term storage. In addition, D‐Fe_3_S_4_ suppository showed comparable therapeutic efficacy with D‐Fe_3_S_4_ in the same infected mouse model, which enhanced the recovery of vaginal tissue by suppressing *G. vaginalis* (Figure 7G,H). Together, these results demonstrated that D‐Fe_3_S_4_ possesses therapeutic characteristics superior than metronidazole for in vivo treatment of bacterial vaginosis.

We further evaluated antibacterial performance of D‐Fe_3_S_4_ using clinical samples from patients affected by bacterial vaginitis. The vaginal secretion from patients was first identified and scored based on the bacterial infection with *G. vaginalis*, *vaginal lactobacillus* or *campylobacter mobilis* using the Nugent score system. The samples with score ≥7 were chosen for antibacterial testing (Table S3, Supporting Information). As shown in Figure 7I, 99.9% *G. vaginallis* were eliminated in the samples of 82 patients. In addition, our treatment also eliminated *Streptococcosis*, *staptococcus*, or *E. coli* infection in the samples of other three patients. However, in the samples of patients carrying *fungifungu*s (11 cases) and *vaginal lactobacillus* (4 cases), our treatment with D‐Fe_3_S_4_ only showed limited antibacterial effects. Together, these results clearly demonstrated that D‐Fe_3_S_4_ is able to effectively eliminate *G. vaginalis* in clinical samples from patients.

## 3 Discussion

In this work, we demonstrated that mFeS materials (e.g., Fe_3_S_4_) are potent antibacterial candidates that kill bacteria in a Gram‐dependent manner, namely by releasing polysulfide species and ferrous iron that exhibit biocidal effects in a highly selective fashion. In particular, the mFeS showed a great potential for bacterial vaginosis treatment for those challenged by resistant *G. vaginosis*. Although BV is treated with a number of effective available antibiotics, such as metronidazole, it has been reported that large number of BV patients experience recurrence within 1 year of treatment for incident disease.^[^ ^31^ ^]^ The major reasons for recurrence are ascribed to the antibiotics resistance and persistence of residual infection. In particular, the persistence may be ascribed to the formation of a biofilm or intracellular strain that protects *G. vaginalis* from antibiotics therapy. Such resistance and persistence are also confirmed in our experimental models. We indeed observed that *G. vaginalis* quickly became tolerant to metronidazole after several passages. Moreover, metronidazole failed to kill the bacteria embedded in biofilm matrix or intracellular vacuole, because it cannot penetrate the barrier to reach the inside bacteria. In contrast, our D‐Fe_3_S_4_ showed high potency against both resistant and persistent *G. vaginosis*, but without causing drug resistance. First, the high antibacterial activity is ascribed to the dual mechanisms of action (MoAs) of D‐Fe_3_S_4_ including polysulfide disrupting glycolysis‐related energy metabolism and iron inducing ferroptotic damage, rendering *G. vaginosis* unable to generate resistance. Second, the antibacterial action mode of D‐Fe_3_S_4_ is releasing polysulfide species and ferrous iron, which can cross the barriers of biofilm matrix and cell membrane as both polysulfides and iron have strong diffusibility In particular, we discovered that the permeability of polysulfide species is strongly correlated with the thickness of bacterial wall, enabling an antibacterial selectivity. Thus, D‐Fe_3_S_4_ not only effectively overcomes metronidazole resistance, but also eliminates persistence strains of *G. vaginosis*, showing superior advantage than the first‐line medication of metronidazole in BV treatment.

In addition, our study provided a readily available candidate of antibiotic alternative as metastable iron sulfides are plenty in nature or can be easily synthesized. There are many iron sulfides present in nature with various interchangeable physical phases including greigite, pyrrhotite, pyrite, marcasite, etc.^[^ ^15^ ^]^ Our study showed that metastable Fe_3_S_4_ exhibited high antibacterial activity, followed byFe_7_S_8_ with limited antibacterial activity, while stable FeS and FeS_2_ (including synthesized FeS_2_ (pyrite) with nanoscale size or microscale size, data not shown) lacked antibacterial activity in our culture assays. The ability of releasing ferrous iron and polysulfide is strongly correlated with the antibacterial activity of iron sulfide minerals. We verified that converting sulfur into iron polysulfides is an effective approach to improve antibacterial performance of sulfur‐containing compounds. We found that hydrothermal method may be an approach to prepare Fe_3_S_4_ with pure phase by supplying organic polysulfide (e.g., DADS) (Figure S1A,B, Supporting Information). Such system also can convert other organosulfurs, sodium sulfides (Na_2_S, Na_2_S_4_) or elemental sulfur into metastable iron sulfides (Fe_3_S_4_, Fe_7_S_8,_ or Fe_1‐_ *_x_* S) (Data not shown). In addition, we found that directly mixing Na_2_S_4_ and FeCl_2_ also forms a colloid (Figure S4I,J, Supporting Information). Although the phase of colloidal product has not yet been clarified, the addition of FeCl_2_ into Na_2_S_4_ increased the production of polysulfides (Figure S4L, Supporting Information) and thus enhanced the antibacterial activity of Na_2_S_4_ (Figure 2J). In addition, phase change is achieved in processed Zirantong, a mineral traditional Chinese medicine, by combining high temperature calcination and vinegar quenching, which can gradually change the phase of pyrite (FeS_2_) to Fe_7_S_8_.^[^ ^32^ ^]^ In addition, many microorganisms, such as magnetotactic bacteria, can synthesize iron sulfide magnetosome containing Fe_3_S_4_ or mackinawite (tetragonal FeS) and cubic FeS which are thought to be precursors of Fe_3_S_4_, providing high quality of iron sulfide with specific crystal and narrow size ranges.^[^ ^33^ ^]^ In view of the abundant resource in geological ore in nature, biomineralization in biological system as well as chemical synthetic technologies, it is possible to prepare metastable iron sulfides at large scale with low cost, which is advantageous for drug development and clinical translation. In particular, these materials can be formulated into powder and suppository for long‐term storage, showing high practicability as medications.

Furthermore, we found that polysulfide species play a bactericidal role by penetrating bacterial wall and inhibiting glycolysis‐related energy metabolism. Our previous studies discovered that polysulfides act as an oxidizer to deplete GSH and thus aggravate redox imbalance in ferrous iron induced ferroptosis‐like death of bacteria under in vitro aqueous conditions.^[^ ^11^ ^]^ Such action of polysulfides was thought to be auxiliary in antibacterial process and the antibacterial efficacy of nFeS toward *S. aureus* decreased dramatically in culture media or in vivo system. In contrast, significant differences of antibacterial activity of Fe_3_S_4_ are evident for these bacteria under culture media with the degree of antibacterial activity changing in the order: *G. vaginalis*>Gram‐negative *E. coli*>Gram‐positive *S. aureus*, showing a strong dependence on the type of bacterial wall, as classified by Gram‐staining (Figure 2A–C). The Gram‐dependent antibacterial mode of polysulfides may explain why nFeS failed to kill *S. aureus* in culture media or physiological conditions. In addition, owing to the selective antibacterial mode, D‐Fe_3_S_4_ showed minimal toxicity to vaginal *lactobacillus* (Gram‐positive species) at the MIC for *G. vaginalis*, making it feasible for combination therapy with D‐Fe_3_S_4_ and probiotic *lactobacillus* for BV. These features render D‐Fe_3_S_4_ superior to other antimicrobial nanomaterials, such as silver nanoparticles which exhibit undifferentiated toxicity to both pathogenic and probiotic bacteria and host cells.^[^ ^34^ ^]^

There are several limitations to our study. First, although the antibacterial mechanism of D‐Fe_3_S_4_ was revealed as targeting to glucokinase in glycolysis, other related pathways may be affected by polysulfide species which induce polysulfidation in molecules containing free thiol group. We will further conduct more detailed analyses of metabolome, transcriptome, and proteome and screen potential targets of polysulfide species in different levels in our future work. Second, there are diverse pathogens in BV progress, and although we determined that D‐Fe_3_S_4_ killed *G. vaginalis* and was safe to vaginal *lactobacillus*, it could be that more pathogens such as *Atopobium vaginae*, *Neisseria gonorrhoeae*, *Mobiluncus spp*., *Bacteroides spp*., and *Prevotella spp*.,^[^ ^35^ ^]^ and fungi (*Candida albicans*),^[^ ^36^ ^]^ might be affected in BV treatment with D‐Fe_3_S_4_. Hence, the broad antimicrobial efficacy will be investigated in our future study to evaluate the potential advantages and limitations of D‐Fe_3_S_4_ in treating BV and restoring microbiota balance.

In conclusion, our data demonstrate that mFeS exhibit unique mechanism different from traditional antibiotics, and the antibacterial action of iron–polysulfide coordination may provide insight for the design of iron‐sulfur antibacterial. Importantly, mFeS represent a promising candidate specifically killing *G. vaginosis* and counteracting drug resistance in BV treatment with high biocompatibility. With the challenge of fast‐evolved resistance in bacteria, mFeS might provide a valuable non‐antibiotic alternative option to help treating BV with broad prevalence and frequent recurrence and improving women's health and life quality.

## 4 Experimental Section

### Materials

All chemicals were of analytical grade. Ethylene glycol, FeCl_3_, NaAc·3H_2_O, FeCl_2_, FeCl_3_, FeS, FeS_2_, diallyl disulfide (DADS), monobromobimane, and lysozyme were purchased from Sigma‐Aldrich. Processed Zirantong was purchased from Kangmei Pharmaceutical Co., Ltd (Sichuan, China). SulfoBiotics‐SSP4, NaHS, Na_2_S, Na_2_S_3_ and Na_2_S_2_ were purchased from DOJINDO (Japan). Na_2_S_4_ was purchased from Shanghai Key Industrial Co. LTD (Shanghai China). Tryptone, brain heart infusion, porcine skin LP0008), yeast extract (LP0021), glucose, starch and drug sensitive piece were purchased from Oxoid (UK). Glutathione (GSH), dimethylsulfoxide (DMSO), and agar were purchased from Sangon Biotech (China). Columbia blood AGAR plate were purchased from Crmicro (Jiangmen, China). Cell Counting Kit‐8, Crystal violet, metronidazole and hydrogen peroxide (H_2_O_2_) were bought from Aladdin Chemistry (Shanghai, China). GSH and GSSG Assay Kit, Enhanced ATP Assay Kit, propidium iodide (PI), 2,7‐dichlorofuorescein diacetate (DCFH‐DA) were purchased from Beyotime Biotechnology (Shanghai, China). SYTO9 green fluorescent nucleic acid stain was purchased from Thermo Fisher Scientific (Waltham, MA). *G. vaginalis* ATCC 14018 were purchased from the American Type Culture Collection (ATCC). *Streptococcus mutans* UA159 (ATCC 700610), *Escherichia coli* (*E. coli*, CMCC(B)44102), *Staphylococcus aureus* (*S. aureus*, ATCC 29213) were purchased from the Institute of Microbiology of the Chinese Academy of Science.

### Ethics Statement

All animal studies were performed following the protocols approved by the Institutional Animal Care and Use Committee of Yangzhou University and the Institutional Animal Care and Use and Committee of the Institute of Biophysics, Chinese Academy of Sciences, respectively. The experimental research for antibacterial test using human vaginal secretion from patients was approved by the Ethics Committee of Xishan People's Hospital of Wuxi City and conducted after obtaining informed consent agreement by all participating patients.

### Synthesis of D‐Fe_3_S_4_ and Characterization

D‐Fe_3_S_4_ synthesis was based on a previously described hydrothermal synthesis method established in the laboratory.^[^ ^11^ ^]^ Briefly, 0.82 g FeCl_3_ was dissolved in 40 mL ethylene glycol, stirred at room temperature for 30 min, and exposed to ultrasound for 10 min to ensure complete dissolving of FeCl_3_. Once the solution was clear, 3.6 g NaAc·3H_2_O was added, stirred at room temperature for 30 min. One mL of diallyl disulfide (DADS) was added with continuous and vigorous stirring for 30 min. The mixture was transferred to a 50 mL Teflon‐lined stainless steel autoclave and reacted at 200 °C for 12 h. After the reaction was completed, the autoclave was cooled to room temperature. The product of black precipitate was washed alternately with H_2_O and ethanol for a total of six times. Next, the products were vacuum‐dried at 60 °C for 5 h. The final product was sealed in tube and placed inside a desiccator for long‐term storage (maximum of 1 month). Morphological and structural characteristics of nFeS were determined using either a transmission electron microscope (TEM, Tecnai G2 F30 S‐TWIN 300 kV), scanning electron microscope (SEM, Hitachi SU‐8010), X‐ray diffractometer (XRD, D8 Advance, Bruker AXS, Germany) or a Zeiss confocal microscope LSM980 (Zeiss LSM980).

### Bacterial Strains and Growth Conditions

*G. vaginalis* ATCC 14018 were obtained from the American Type Culture Collection (ATCC), vaginal clinical isolates were obtained from vaginal swabs from the Department of Microbiology, Xishan People's Hospital, Wuxi city (based on Nugent score, BV ≥ 7 points). The swabs were immediately used for inoculation on a Columbia blood agar plate, and the bacteria were cultured under anaerobic ambient at 37 ℃ with 5% CO_2_ for 48 h. Finally, candidate *Gardnerella* species were identified by stroma‐assisted laser desorbed/ionization time‐of‐flight (MALDI‐TOF, Bruker Daltonics, Mabilika, USA). *G. vaginalis* was cultured in BHI media composed of brain/heart infusion broth containing 1% gelatin, 1% yeast extract, 0.1% soluble starch, 0.1% glucose and 10% fetal bovine serum. *Lactobacillus vaginalis* was isolated from vaginal secretions of healthy women and screened for antibacterial activity against *G. vaginalis*, and production of hydrogen peroxide, lactic acid and form biofilm, *vaginal lactobacillus* was cultured in MRS broth. Agar plates and liquid cultures were incubated at 37 ℃ with 5% CO_2_, frozen stocks of strains were stored at −80 ℃ in BHIs containing 30% (v/v) glycerol.

### Identification of Polysulfide and Iron Released from D‐Fe_3_S_4_

The polysulfide species released by D‐Fe_3_S_4_ were identified by liquid chromatography‐mass spectrometry triple TOF 5600 plus. Briefly, 30 µL supernatant of D‐Fe_3_S_4_ (1 mg mL^–1^) was added into a tube containing 70 µL 100 × 10^‐3^ m Tris–HCl buffer (pH 9.5, with 0.1 × 10^‐3^ m diethylenetriaminepentaacetic acid (DTPA)), and then 50 µL monobromobimane (mBBr) (10 × 10^‐3^ m, in acetonitrile solution) was added and incubated at 25 ℃ for 30 min, followed by adding 50 µL of 5‐sulfosalicylic acid (200 × 10^‐3^ m). The pretreated samples were injected into a reverse phase symmetry C18 column (ACQUITY UPLC CSH C18: 2.1 × 100 mm, 1.7 µm particle size, Waters). The mobile phases were A (0.1% FA in water) and B (acetonitrile in 0.1% FA), and the flow rate was 0.25 mL min^‐1^. The samples were separated with a gradient as follows: 0–1 min, 15%B; 1–10 min, 15%–45% B; 10–11 min, 45%–95% B; 11–12 min, 95%–15% B; 13–18 min, 15% B and hold for 5 min. The mass range parameters were as follows: CE 10.000; DP 100.000; IDIx 0.000; IDUx 5.000; IRDx 15000.000; IRWx 10000.000; IWIx 0.000; IWUx 5.000; XA1 136.617; Start Mass:50.0; End Mass: 1000.0. The effluent was then applied to tandem mass spectrometer using an electrospray ionization (ESI) interface, and operated in the positive‐ion mode. The results were monitored in multiple reaction monitoring mode. The iron released from D‐Fe_3_S_4_ was measured using a Serum Iron Concentration Assay kit (Solarbio, Beijing, China).

### Preparation of Vaginal D‐Fe_3_S_4_ Suppository

Suppository formulations^[^ ^37^ ^]^ were prepared with the following percentage by mass: 20% gelatin, 70%glycerol, 9.9% distilled water, 0.1% D‐Fe_3_S_4_. Gelatin was soaked in a few mL of water until it became swollen and softened. Glycerol was then added and mixed well. Next, D‐Fe_3_S_4_ was added and mixed well and the mixture was heated to 80 ℃ with continuously stirring for 4 h. After standing at 75 ℃ for 1 h, the mixture was injected into the lubricant‐coated and pre‐cooled mold. After cooling down, the black, translucent and elastic suppository was formed and collected for experimental use.

### In Vitro Antibacterial Assays

Five to seven colonies of *G. vaginalis* on Columbia blood agar plates were randomly selected and inoculated into 5 mL of BHIs culture and was cultured at 37 ℃ with 5% CO_2_ for 12–18 h. One day post‐inoculation, required amounts of *G. vaginalis* bacterial solution was taken and transferred into fresh medium at a ratio of 1:100 and cultured at 37 ℃ with 5% CO_2_ for 6–8 h. When the OD_600_ reached a value of 0.5, 100 µL of bacterial inoculation was mixed with BHIs (900 µL) as the control. An additional 100 µL of bacterial inoculation was mixed with D‐Fe_3_S_4_ and BHIs (800 µL) as the experimental group. After incubation at 37 ℃ for certain time, bacterial viability was checked by plating bacteria with proper dilution and calculating the bacterial number in CFU mL^–1^. The inhibition assays using either FeCl_2_, Na_2_S_4_, EDTA, ferrostatin‐1 or other inhibitors were conducted in the same system and using the same procedure. Glutathione levels were measured using GSH and GSSG assay kits (Beyotime, China). The fluorescent probe Bodipy581/591‐C11 was used to measure the lipid peroxidation level of bacteria. ATP Content Assay Kit and Micro Glucokinase (GLK) Assay Kit (Solarbio, China) were used to measure ATP and glucokinase activity levels.

### Intracellular Antibacterial Assays

VK2 cells were cultured to exponential phase and divided into six well plate with complete DMEM medium (90% DMEM and 10% FBS without antibiotics) with 80–90% coverage of plate surface at 37 °C with 5% CO_2_. In parallel, *G. vaginalis* were cultured, collected and suspended into DMEM medium at 5 × 10^8^ CFU mL^‐1^. *G. vaginalis* infected VK2 cells for 3 h at 37 ℃ with 5% CO_2_. After discarding the supernatant and washing twice with PBS, the extracellular bacteria were killed with clindamycin (0.1 µg mL^–1^) for 3 h. 1024 × 10^‐6^ m metronidazole and D‐Fe_3_S_4_ was then introduced to kill intracellular bacteria by 3 h incubation at 37 ℃ with 5% CO_2_. A sample of the cells was lysed using buffer containing 0.1% Triton‐X. The number of viable bacteria was counted by plating serial dilutions of bacteria on Columbia blood agar plate.

### Drug Sensitivity Test

The *G. vaginalis* isolates were evaluated for in vitro antimicrobial susceptibilities to metronidazole, D‐Fe_3_S_4_, Ag NPs, FeCl_2_, NaHS, Na_2_S, Na_2_S, Na_2_S_2_, Na_2_S_3_, Na_2_S_4_ and EDTA using the Broth dilution method previously described by the Clinical and Laboratory Standards Institute. When the *G. vaginalis* culture was determined to be at its logarithmic growth stage, (OD_600_ = 0.5), containing approximately 1–2 × 10^8^ CFU mL^–1^, *G. vaginalis* was diluted 100 times in BHI solution, to approximately 1–2 × 10^6^ CFU mL^–1^. Next, different concentrations of drugs were added to the prepared bacterial suspension. After cultured for 24 h, the end reading of the OD_600_ point was measured with a microplate reader to evaluate the growth of bacteria. The minimum inhibitory concentration (MIC) was defined as the lowest antibiotic concentration at which growth is significantly reduced or no growth at all.

### RNA‐Seq for Transcriptome Analysis and Metabolome Analysis

Five to seven colonies of *G. vaginalis* on Columbia blood agar plates were randomly selected and inoculated into 5 mL of BHIs culture, and *G. vaginalis* was cultured at 37 ℃ with 5% CO_2_ for 12–18 h. One day post inoculation, 0.5 mL of *G. vaginalis* bacterial solution were transferred into the fresh medium at a ratio of 1:100 and cultured at 37 ℃ with 5% CO_2_ for 6–8 h. When the OD_600_ reached a value of 0.8, 900 µL of the bacterial solution was mixed with either 100 µL BHIs (Control) or 100 µL Na_2_S_3_, Na_2_S_4_, D‐Fe_3_S_4_ (100 × 10^‐6^ m) and incubated at 37 ℃ with 5% CO_2_ for 60 min. Following drug treatment and washing the *G. vaginalis* bacteria cells twice with PBS, the supernatants were completely removed following centrifugation at 4 ℃ at 10 000 rpm for 2 min and the bacteria pellets were collected. After washing twice with PBS, the *G. vaginalis* pellets were immediately placed into liquid nitrogen and frozen for 15 min. After freezing, the *G. vaginalis* was stored at ‐80 ℃. Bacteria were processed for transcriptome analysis and metabolome analysis. Total RNA extraction, RNA sequencing and bioinformatic data collection were performed by Shanghai Personalbio (Shanghai, China).

### Molecular Dynamic Simulation for Glucose Interaction with Glucokinase

The energy profile for the insertion reaction of polysulfide (the HS_3_^−^ was used for calculation based on the estimated pKa value of oligosulfanes ^[^ ^38^ ^]^) and Cys in water was calculated using the B3LYP functional ^[^ ^39^ ^]^ in conjunction with the 6‐31G* basis sets^[^ ^40^ ^]^ integrated in the Gaussian09 package.^[^ ^41^ ^]^ For the molecular dynamic (MD) simulations, the initial conformations for the complexes of glucokinase and glucose were obtained by docking using the AutoDock Vina package.^[^ ^42^ ^]^ For each complex, five conformations with top 5 docking scores were dissolved in water for 20 ns MD simulations. To quantify the strength of the interaction between glucokinase and glucose, the interaction energy (*E*_int_ in kJ mol^–1^) including the short‐range Coulombic interaction (*E*_Coul_) and the short‐range Lennard‐Jones energy (*E*_LJ_) were calculated. The conformations with the most negative *E*_int_ were selected for further investigations. All the MD simulations and analysis were performed by GROMACS software.^[^ ^43^ ^]^

### Vaginalis Biofilm Formation

Cell slides were placed at the bottom of the 24‐well sterile plate and incubated with 2.5 mL BHIs for 30 min. Then the culture solution was exchanged with 2.5 mL of prepared bacterial suspension (approximately 10^6^ CFU mL^–1^) in the BHIs with 0.1% (w/v) glucose per well and incubated at 5% CO_2_, 37 ℃ for 24 h. The planktonic cells were carefully removed, and 2.5 mL of fresh medium was added to each well before the plates were further incubated for 24 h. After 48 h of culturing, the biofilms were treated with D‐Fe_3_S_4_ for 3 h. The biofilms were then washed once with PBS. Either 24 or 48 h monospecies biofilm of *G. vaginalis* was used as a control. BHI solution was used as a negative control in all experiments to exclude any possible contamination of bacteria. The biofilms were collected for dry weight measurement, viability assay of *G. vaginalis*.

The dual‐species biofilm of *G. vaginalis* and *lactobacillus* was established using similar procedure. Briefly, following 24 h of *G. vaginalis* biofilm formation, the planktonic cells remaining in the solution were carefully removed, before 2 mL of fresh BHI solution was added to each well. Simultaneously, the suspension of *lactobacillus* was added (10^6^ CFU mL^–1^) to each well and the plates were further incubated for 24 h. Next, the dual‐species biofilms were washed once with phosphate buffer saline (PBS) solution. All assays were repeated three times with five technical replicates.

### SEM and Confocal Microscopy for *G*. *Vaginalis* Biofilms

The structure of *G. vaginalis* biofilm treated by D‐Fe_3_S_4_ was examined by scanning electron microscope (SEM). First, *G. vaginalis* biofilms were resuspended in glutaraldehyde (2.5%, Sigma‐Aldrich) for 24 h at 4 °C under dark conditions. Bacterial cells were then washed and treated with ethanol gradient dehydration (30%, 50%, 70%, 90%, and 100% twice), before being dried using a critical point dryer and coated with platinum sputter. Finally, scanning electron microscope (SEM) images were obtained on a Hitachi S‐4800 FE‐SEM at a working voltage of 15.0 kV and a working current of 10 µA under magnification of 40 K. The 3D structure of biofilm was also characterized using confocal microscopy. Once the biofilm were grown, 1 µg mL^‐1^ PI and 5 × 10^‐6^ m Syto9 staining solution were added to the surface of the biofilm and incubated for 20 min avoiding light. Next, the samples were washed twice with PBS before being analyzed using laser confocal microscope FV1000 (OLYMPUS FV1000).

### Mouse Vaginal Infection Model

Seven week old female Balb/C mice were obtained from the Institute of Biophysics, Chinese Academy of Sciences, Beijing. Mice were inoculated vaginally with 1 × 10^8^ CFU *G. vaginalis* (ATCC14018) in 10 µL sterile PBS, and inoculated for three consecutive days. Three days of post infection, the mice were divided into three groups (five mice per group). The infected mice in the different groups were treated with 10 µL PBS, 500 μM D‐Fe_3_S_4_, 500 μμ metronidazole for 72 h, respectively. Vaginal washes were collected by flushing vaginas with 10 µL sterile PBS using a P10 pipet, followed by rinsing into an additional 90 µL PBS in a sterile 1.5 mL Eppendorf tube. *G. vaginalis* titers were determined from washes by preparing 10‐fold serial dilutions in PBS (in the anaerobic chamber), before spotting 100 µL of each dilution onto Columbia blood agar plates. Colonies were then enumerated and reported as recovered colony forming units (CFU) per mL of vaginal fluid. The vaginal orifices of mice were photographed. The mice were sacrificed 7 d postinfection, and the vagina, uterus, heart, liver, spleen, lung, kidney, and tissue were collected and fixed with formalin. Tissues were then dissected and analyzed by hematoxylin‐eosin (HE) staining. The tissue sections were examined using a Nikon Eclipse CI microscope in bright field mode.

Another infection model was established using clinical metronidazole‐resistant strain MRGV with similar infection procedure as above. Mice successfully infected with MRGV were then randomly divided into 5 groups (five mice per group), 1: Control, 2: D‐Fe_3_S_4_, 3: Metronidazole, 4: LV, 5: D‐Fe_3_S_4_+ LV. Mice in groups 1–3 were inoculated with PBS, and groups 4 and 5 were inoculated with 1 × 10^8^ CFU mL^–1^ *Lactobacillus vaginalis* with 10 µL sterile PBS at 8:00 am. Groups 1 and 4 were treated with PBS, groups 2, 3 and 5 were treated with D‐Fe_3_S_4_ (3.38 × 10^‐3^ m), metronidazole (3.38 × 10^‐3^ m) and D‐Fe_3_S_4_ (3.38 × 10^‐3^ m) at 8:00 pm, respectively. The treatment was performed for 3 d and all mice were sacrificed for bacterial viability assay.

### D‐Fe_3_S_4_ to Eliminate Bacteria in Clinical Samples

The clinical samples were collected from 100 women from the Department of Obstetrics and Gynecology of Wuxi Xishan People's Hospital. This study was approved by the Ethics Committee of Wuxi Xishan People's Hospital (approval number: LLS2020KY036 27/07/2020). All samples were examined by Gram staining and microscopy to assess their Nugent score, patients with Nugent score ≥ 7 were selected. Vaginal secretion was collected with a sterile cotton swab and then resuspended with 1 mL water. After mixed thoroughly, 0.5 mL of suspension was added D‐Fe_3_S_4_, the left was taken as the control. After 3 h incubation at room temperature, colonies counting of microbes were conducted and presented in CFU mL^–1^.

### Biosafety Assessment of D‐Fe_3_S_4_

The cytotoxicity of D‐Fe_3_S_4_ was evaluated in VK2 cells (ATCC 2616) using CCK8 kit. VK2 cells were inoculated into a sterile 96‐well plate (Corning, NY) and incubated in DMEM with 10% FBS under 5% CO_2_ at 37 °C for 12 h. The initial concentration of the VK2 cells was 5000 × 10^4^ cells per well. VK2 cells were treated with a series of concentrations of D‐Fe_3_S_4_ (64 × 10^‐6^, 128× 10^‐6^, 256 × 10^‐6^, 512 × 10^‐6^ and 1024 × 10^‐6^ m) for 24 h. Finally, CCK8 was added to a 96‐well plate and incubated with VK2 cells for 2–3 h. The supernatant were extracted, and measured at 532 nm using a miniature tablet reader. Relative cell viability was calculated by comparing with untreated control cells. The cytotoxicity of the supernatant containing polysulfide species and ferrous iron released from D‐Fe_3_S_4_ was evaluated with the VK2, macrophages (RAW264.7) and HaCaT (human keratinocytes) using the above same procedure. The supernatant was prepared by incubating D‐Fe_3_S_4_ in water for 24 h at room temperature and collected via a centrifugation at 10 000 g for 10 min.

For animal test, ten healthy Balb/C mice (7 week old female mice) were randomly divided into two groups with five mice in each group. 10 µL (1024 × 10^‐6^ m) D‐Fe_3_S_4_ was injected into the vagina in one group and PBS was injected into the mice of the control group. Vaginal inoculation was performed once a day for seven days for each mouse. The mice's behavior, including feeding, drinking, activity, and weight, was then continuously observed and recorded. Finally, three mice in each group were sacrificed for vaginal histopathological examination and major organ histochemical examination.

### Statistical Analysis

Each experiment was repeated at least three times and the data were interpreted as means ± SD. GraphPad Prism8.3 was used for statistical analysis. The t‐test was used to make a comparison between the two sides (test and control). The statistical significance of the data in the present study was expressed as *p < 0.05, **p < 0.01, ***p < 0.001, and****p < 0.0001).

## Conflict of Interest

The authors declare no conflict of interest.

## Author Contributions

L.F. and R.M. contributed equally to this work. L.G. conceived and organized the project and wrote the manuscript. L.F. and Q.W. conducted in vitro and in vivo antibacterial tests of iron polysulfide. Y.L. and X.W. helped synthesis and characterization of iron sulfide minerals. L.C. conducted theoretical analysis of Fe–S bonds and X.J.G. analyzed the binding of glucokinase with glucose. Y.H. and T.W. performed structure prediction of glucokinase and purified glucokinase. H.W., Q.S., and C.C. provided clinical guideline and samples. J.J. guided the cell and animal tests. L.G. contributed to data discussion and drafted the manuscript. All authors contributed to the editing of the manuscript.

## Supporting information

Supporting Information

Supporting Information

Supporting Information

## Data Availability

The data that support the findings of this study are available from the corresponding author upon reasonable request.
