# Supplementary material for: Metastable Iron Sulfides Gram‐Dependently Counteract Resistant Gardnerella Vaginalis for Bacterial Vaginosis Treatment
Source: Adv Sci (Weinh). 2022 Feb 5;9(10):2104341. doi: 10.1002/advs.202104341 (PMC8981900; doi:10.1002/advs.202104341)
Supplement: Supplementary file 1 — Supporting Information [file ADVS-9-2104341-s002.pdf]

ADVANCED SCIENCE
Open Access

Supporting Information

for *Adv. Sci.*, DOI 10.1002/advs.202104341

Metastable Iron Sulfides Gram-Dependently Counteract Resistant *Gardnerella Vaginalis* for Bacterial Vaginosis Treatment

*Ling Fang, Ruonan Ma, Xuejiao J. Gao, Lei Chen, Yuan Liu, Yanwu Huo, Taotao Wei, Xiaonan Wang, Qian Wang, Haojue Wang, Chengjun Cui, Qifeng Shi, Jing Jiang and Lizeng Gao**

ADVANCED SCIENCE
Open Access

Supporting Information

for *Adv. Sci.,* DOI: 10.1002/advs.202104341

Metastable Iron Sulfides Gram-Dependently Counteract Resistant *Gardnerella Vaginalis* for Bacterial Vaginosis Treatment

*Ling Fang, Ruonan Ma, Xuejiao J. Gao, Lei Chen, Yuan Liu, Yanwu Huo, Taotao Wei, Xiaonan Wang, Qian Wang, Haojue Wang, Chengjun Cui, Qifeng Shi, Jing Jiang, Lizeng Gao**

Supporting Information

Metastable Iron Sulfides Gram-Dependently Counteract Resistant *Gardnerella Vaginalis* for Bacterial Vaginosis Treatment

*Ling Fang, Ruonan Ma, Xuejiao J. Gao, Lei Chen, Yuan Liu, Yanwu Huo, Taotao Wei, Xiaonan Wang, Qian Wang, Haojue Wang, Chengjun Cui, Qifeng Shi, Jing Jiang, Lizeng Gao**

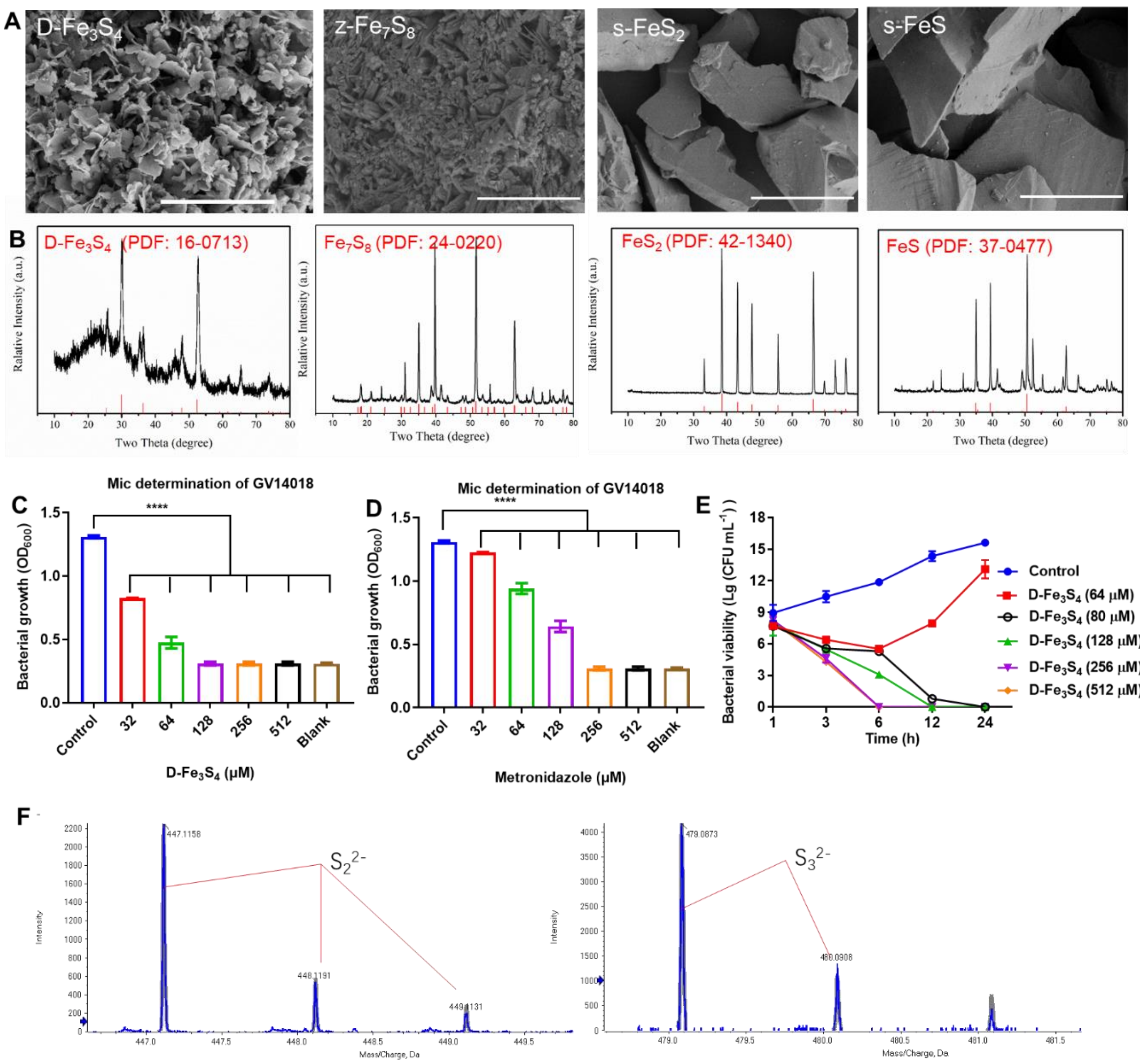

Figure S1. Identification, antibacterial activity and release of ferrous iron and polysulfide species of iron sulfides. **A**) SEM characterization of four typical iron sulfides for antibacterial screening. SEM image showing the morphology of the iron sulfide crystals. Left side of the two-scale bar: 5 μm. Right side of the two-scale bar: 20 μm. **B**) XRD characterization of D-Fe_3S_4**,** z-Fe_7S_8, s-FeS_2 and s-FeS. **C**) Antibacterial activity of D-Fe_3S_4 toward *G. vaginalis* (in BHIs). **D**) Antibacterial activity of metronidazole toward *G. vaginalis* (in BHIs). **E**) Dosage and time dependence of antibacterial activity of D-Fe_3S_4 (in BHIs). **F)** HPLC-MS analysis of polysulfides in the supernatant of D-Fe_3S_4 dissolved in water for 1 h. (All sulfides reacted with MBBr). ****p < 0.0001. All experiments were performed in triplicate, and the representative images are shown. Mean±SD are shown.

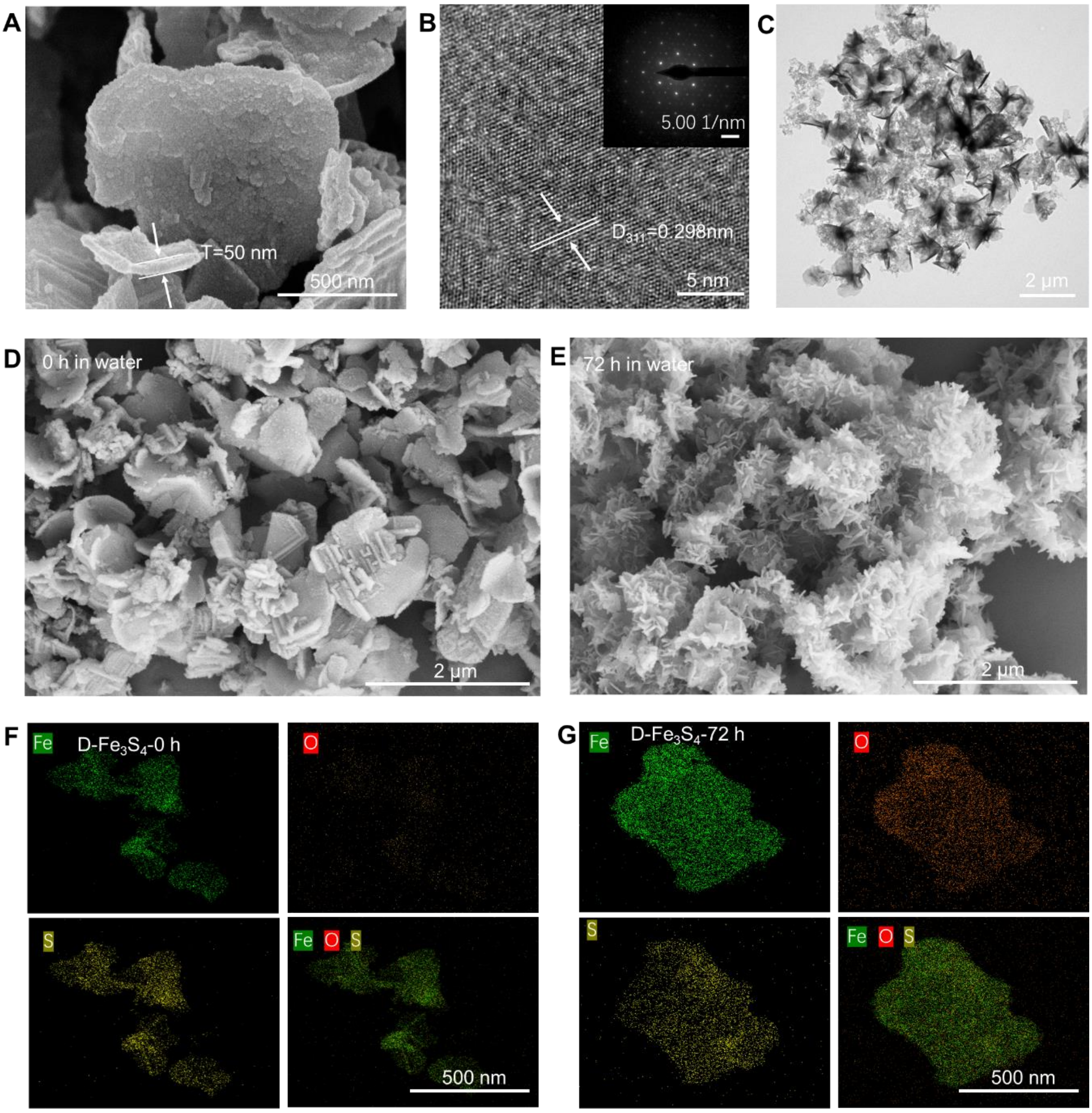

H

Samples	S		Fe		O	
	wt%	Atom%	wt%	Atom%	wt%	Atom%
D-Fe_3S_4-0 h	36.37	37.36	46.53	27.44	17.10	35.20
D-Fe_3S_4-72 h	11.91	9.20	41.26	18.30	46.83	72.50

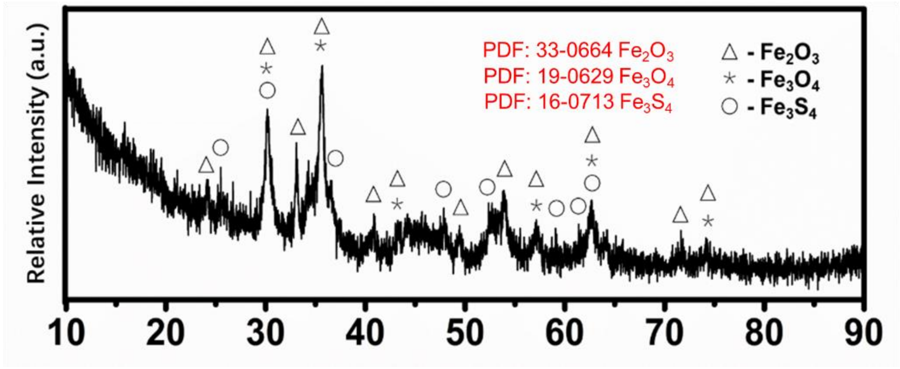

Figure S2. Characterizations of D-Fe_3S_4 and structure transformation when releasing polysulfide species. **A**) SEM characterization of D-Fe_3S_4 demonstrated a nanosheet-like structure with a thickness at about 50 nm. T=50: Thickness at 50 nm. Scale bar: 500 nm. **B**) High-resolution SEM image demonstrated the distance of crystal lattice at 0.298 nm matching D311 facet of D-Fe_3S_4. The insert was the analysis by X-ray single crystal diffraction and indicated the single crystalline of D-Fe_3S_4. **C**) TEM image confirmed the nanosheet structure of D-Fe_3S_4. Scale bar: 2 µm. **D**) SEM image of D-Fe_3S_4 dissolved in water for 0 h. Scale bar: 2 µm. **E**) SEM image of structure change of D-Fe_3S_4 dissolved in water for 72 h. Scale bar: 2 µm. All experiments were performed in triplicate, representative results are shown. **F-H**) EDS analysis of the change of elements (Fe, S, O) in D-Fe_3S_4 in the process of polysulfide species**.** Both ratios of mass (wt%) and atoms (atom%) were shown in the table (H). **I**) XRD analysis of Fe_3S_4 after 72h incubation in water. XRD patterns of D-Fe_3S_4 precipitate after 72 h incubation in water and possible substances in the precipitate analyzed with JADE6 software. Noted: due to low peaks and high noise, such XRD patterns only showed possible existence of iron oxide substances such as Fe_2O_3 or Fe_3O_4. All experiments were performed in triplicate, representative results are shown.

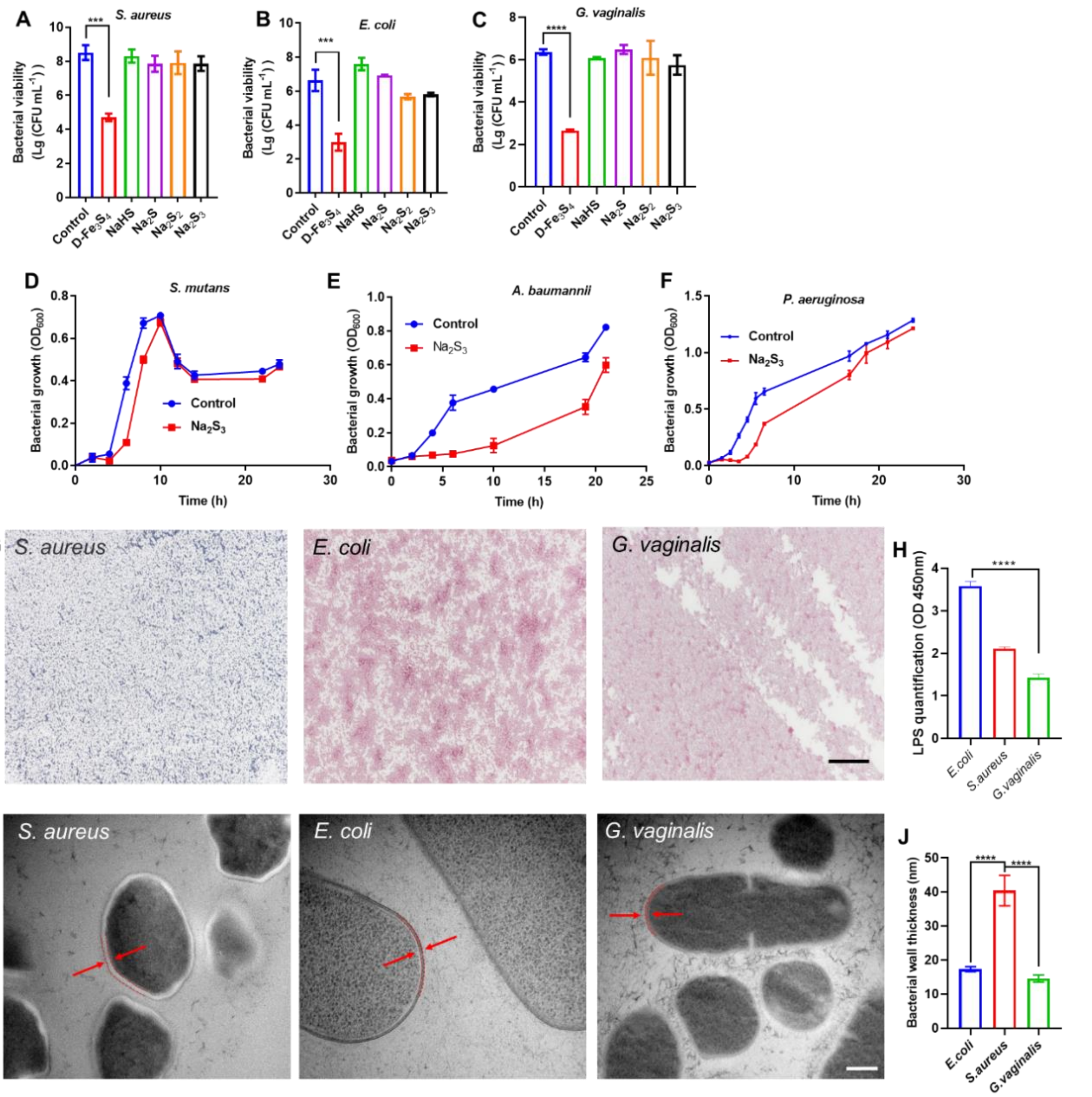

Figure S3. Antibacterial activity of polysulfide species and bacterial wall characterizations. **A)** Antibacterial activity of polysulfide species to *S. aureus* in water. **B**) Antibacterial activity of polysulfide species to *E. coli* in water. **C**) Antibacterial activity of polysulfide species to *G. vaginalis* in water. **D)-F**) Antibacterial activity of Na_2S_3 to *S. mutans* (D). *A. baumannii* (E) and *P. aeruginosa* (F**)**. **G**) Gram staining for bacteria *S. aureus*, *E. coli* and *G. vaginalis*. Scale bars: 100 µm. Representative images are shown. **H**) Quantitative measurement of LPS in the above three bacteria. Scale bar: 200 nm. **I**) Bacterial wall characterization of *S. aureus*, *E. coli* and *G. vaginalis* using high resolution TEM. **J**) Quantitative measurement of bacterial walls based on TEM images in (I). $n = 3$, $***p < 0.001$, $****p < 0.0001$. All experiments were performed in triplicate, representative results are shown. Mean±SD are shown.

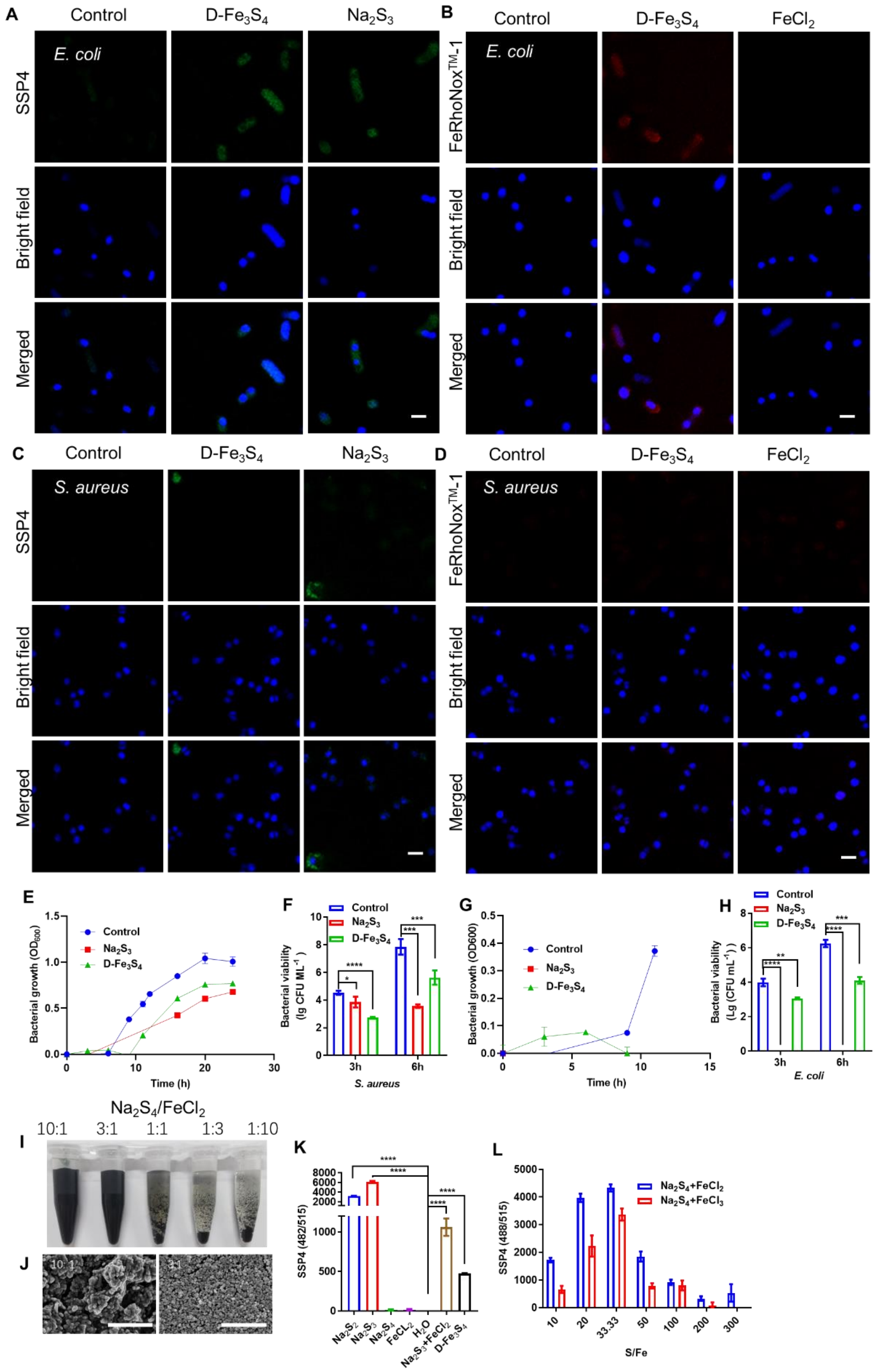

Figure S4. Accessibility of polysulfide to bacteria and enhanced antibacterial performance in the presence of lysozyme or iron. **A**) Confocal imaging of SSP4 as the probe in *E. coli* treated

by D-Fe_3S_4 or Na_2S_3. Scale bars: 1 µm. **B**) Confocal imaging of FeRhoNoxTM-1 as the probe in *E. coli* treated by D-Fe_3S_4 or $FeCl_2$. Scale bars: 1 µm. **C**) Confocal imaging of SSP4 as the probe in *S. aureus* treated by D-Fe_3S_4 or Na_2S_3. Scale bars: 1 µm. **D**) Confocal imaging of FeRhoNoxTM-1 as the probe in *S. aureus* treated by D-Fe_3S_4 or $FeCl_2$. Scale bars: 1 µm. **E**) and **F**) Antibacterial activity of polysulfide species to lysozyme digested *S. aureus*. **G**) and **H**) Antibacterial activity of polysulfide species to lysozyme digested *E. coli*. **I**) Mixtures of different concentrations of Na_2S_4 and ferrous chloride form black colloidal solutions or black precipitates. When the molar concentration of polysulfide is greater than that of iron, the black colloid solution is formed. When the molar concentration of polysulfide is less than that of iron, black precipitates are formed. **J**) The colloid contains nanoparticles of iron sulfide. Scale bar: 500 nm. **K**) SSP4 probe detection (S_3^{2-} signal) of polysulfide concentration from polysulfide standards and the mixture of iron and sodium polysulfides. **L**) The introduction of ferrous iron dramatically increased the S_3^{2-} signal of Na_2S_4. *$p < 0.05$, **$p < 0.01$, ***$p < 0.001$ and ****$p < 0.0001$. All experiments were performed in triplicate, and representative results are shown. Mean±SD are shown.

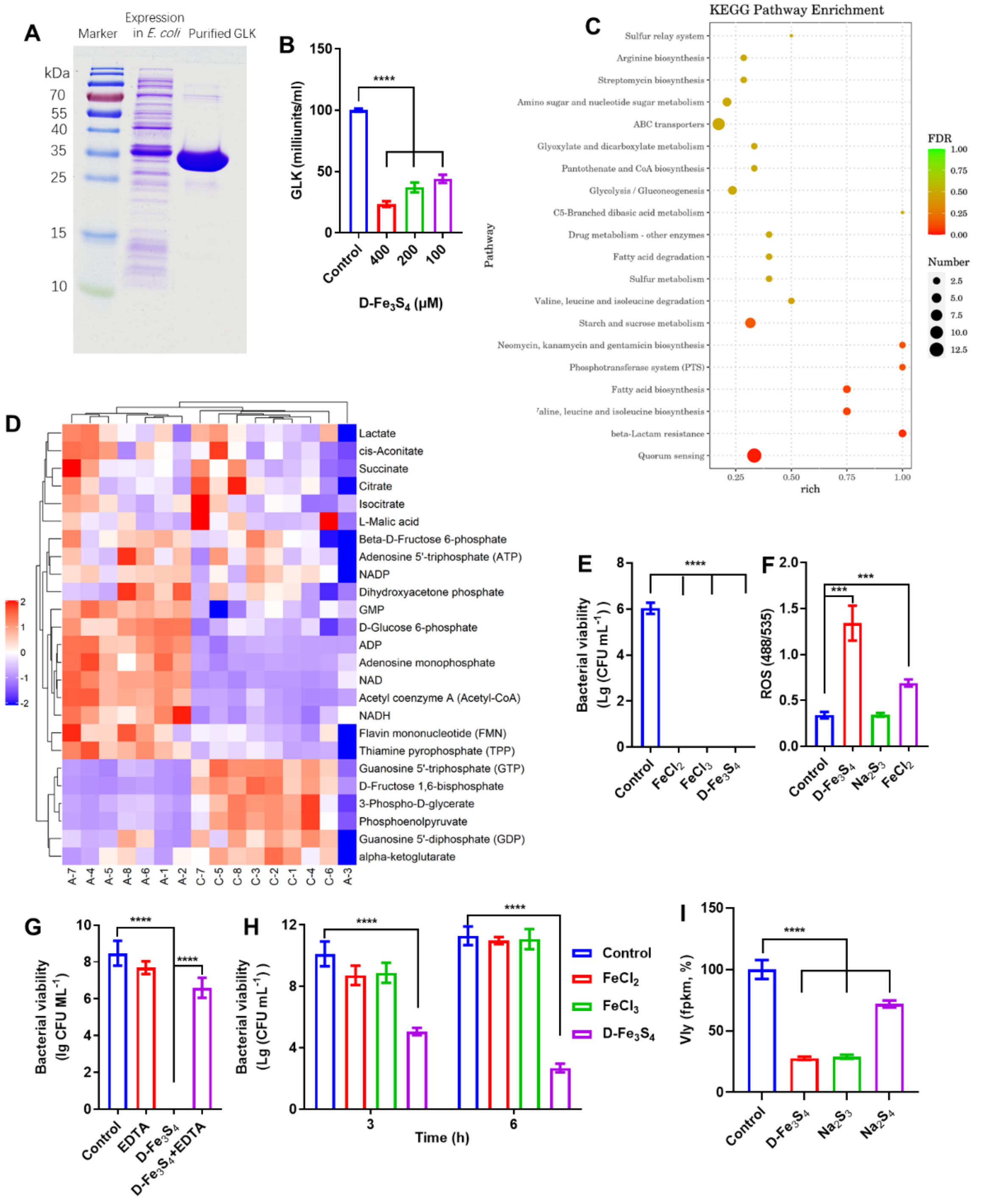

Figure S5. Mechanism analyses of D-Fe_3S_4 against *G. vaginalis*. **A**) *G. vaginalis* glucokinase purified from *E. coli* expression system. The molecular weight of purified GLK (with his-tag) was approx. 34 kDa. **B**) Influence of different concentrations of D-Fe_3S_4 on glucokinase activity in the lysate of *G. vaginalis*. **C**) KEGG pathway enrichment in transcriptome analysis of *G. vaginalis* treated by D-Fe_3S_4 (vs control). **D**) The heatmap of metabonomics analysis showing that abnormal change of intermediates occurred in the glycolytic pathway in *G. vaginalis*. A is control group and C is Na_2S_3 treated group (n=8). **E**) Antibacterial activity of D-Fe_3S_4 and iron to bacteria in water. **F**) Lipid peroxidation of bacteria incubated with D-

Fe_3S_4 or iron in water. **G**) EDTA can reverse the killing of D-Fe_3S_4 by chelating iron. **H**) When incubating bacteria with D-Fe_3S_4 in culture media, D-Fe_3S_4 rather than ferrous or ferric iron can suppress *G. vaginalis*. **I**) Influence of polysulfide species and D-Fe_3S_4 on transcription of vaginolysin (Vly). $^{**}p < 0.01$, $^{***}p < 0.001$, $^{****}p < 0.0001$. All experiments were performed in triplicate, and representative results are shown. Mean±SD are shown

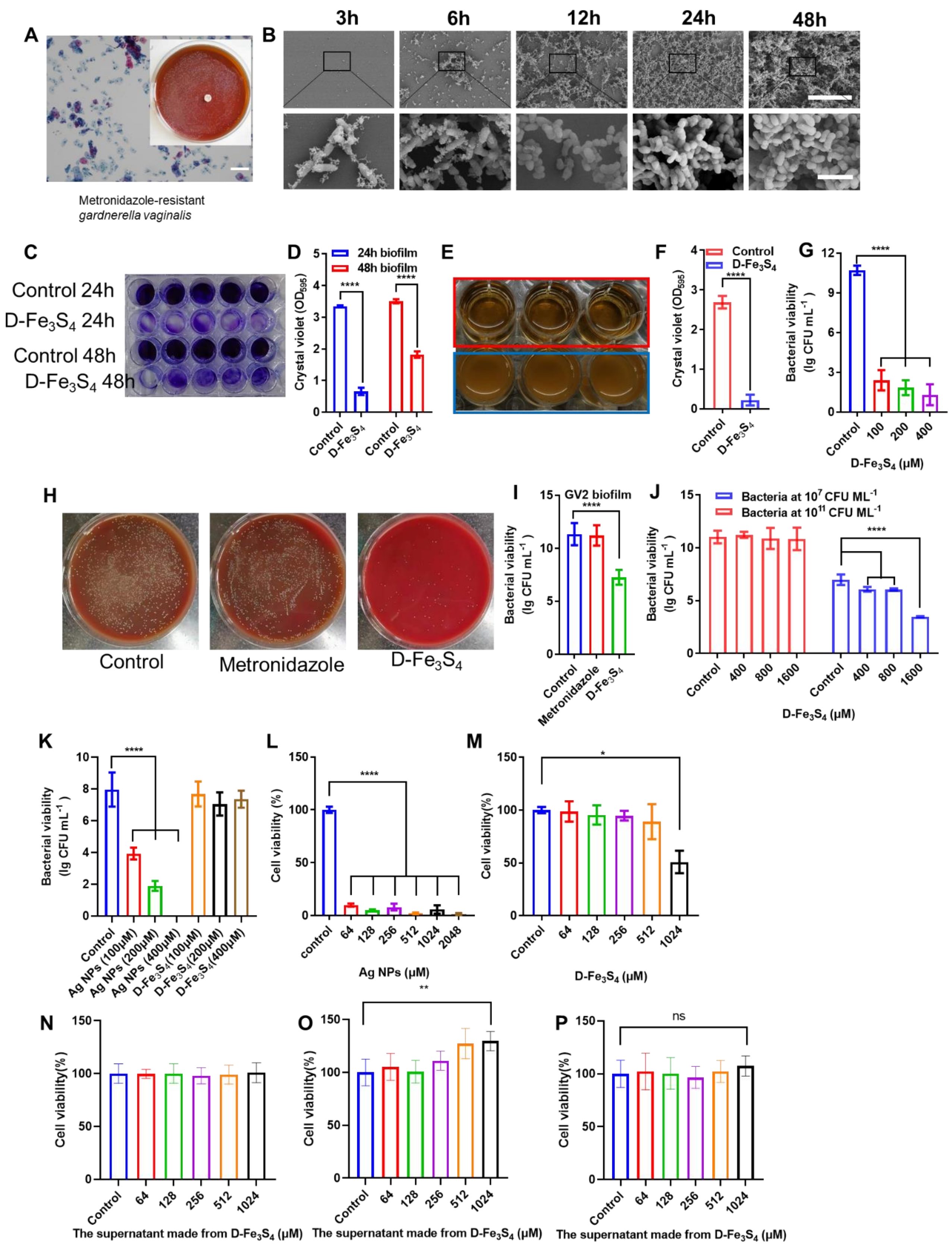

Figure S6. D-Fe_3S_4 overcomes resistance of *G. vaginalis* and is biocompatible to probiotics and mammal cells. **A**) Metronidazole-resistant *G. vaginalis* screened from clinical sample. Clue cells are the normal squamous epithelial cells infected by *G. vaginalis* or *microbacillus*, so that the normal epithelial cell morphology changes, such as irregular edges, rough, not high

transparency. It is a morphological manifestation of a large number of anaerobes such as *katnaerobacteria* attached to exfoliated vaginal epithelial cells. Scale bar: 50 μm. The test for metronidazole-resistance of *G. vaginalis* showed the white dot that indicates *G. vaginalis* is metronidazole resistant. **B**) *G. vaginalis* biofilm formation process: 3h-6h-12h-24h-48h. First row scale bar: 50 μm; second row scale bar: 2 μm. **C**) and **D**) Crystal violet staining of 24 h and 48 h mature *G. vaginalis* biofilm treated with D-Fe_3S_4. **E**)-**G**) D-Fe_3S_4 was added in the process of biofilm formation. The biofilms almost stopped growing in the following 24 h incubation. Such strong inhibition was demonstrated by crystal violet staining (F) and CFU counting assay (G). **H**) and **I**) Metronidazole resistant strain of *G. vaginalis* can be effectively suppressed by D-Fe_3S_4 in the biofilm. **J**) D-Fe_3S_4 cannot kill *vaginal lactobacillus* under *in vitro* aqueous condition (water) for 3 h incubation. **K**) The comparison of the biosafety between D-Fe_3S_4 and silver nanoparticles (Ag NPs). Silver nanoparticles showed stronger suppression on *vaginal lactobacillus* with MIC at 256 µM. 4-log reduction of *vaginal lactobacillus* was caused by 100 µM silver nanoparticles under *in vitro* aqueous condition (water) for 1 h incubation, while D-Fe_3S_4 showed limited antibacterial effect to *lactobacillus* under same condition. **L**) Cytotoxicity assay showed that silver nanoparticles at 64 µM killed more than 90% VK2 cells. **M**) Cytotoxicity assay of D-Fe_3S_4 to mammal cells. VK2 cells remained 89.03% after D-Fe_3S_4 at 512 µM. **N-P**) Cytotoxicity assays of the supernatant containing polysulfide species and ferrous iron released from D-Fe_3S_4 to normal mammal cells. The supernatant showed no cytotoxicity to VK2 (an epithelial cell line from normal vaginal mucosal tissue) (N), RAW264.7 (macrophages from mouse) (O) and HaCaT (the immortalized human keratinocytes) (P).*$p < 0.05$ and ****$p < 0.0001$. All experiments were performed in triplicate, and representative results are shown. Mean±SD are shown.

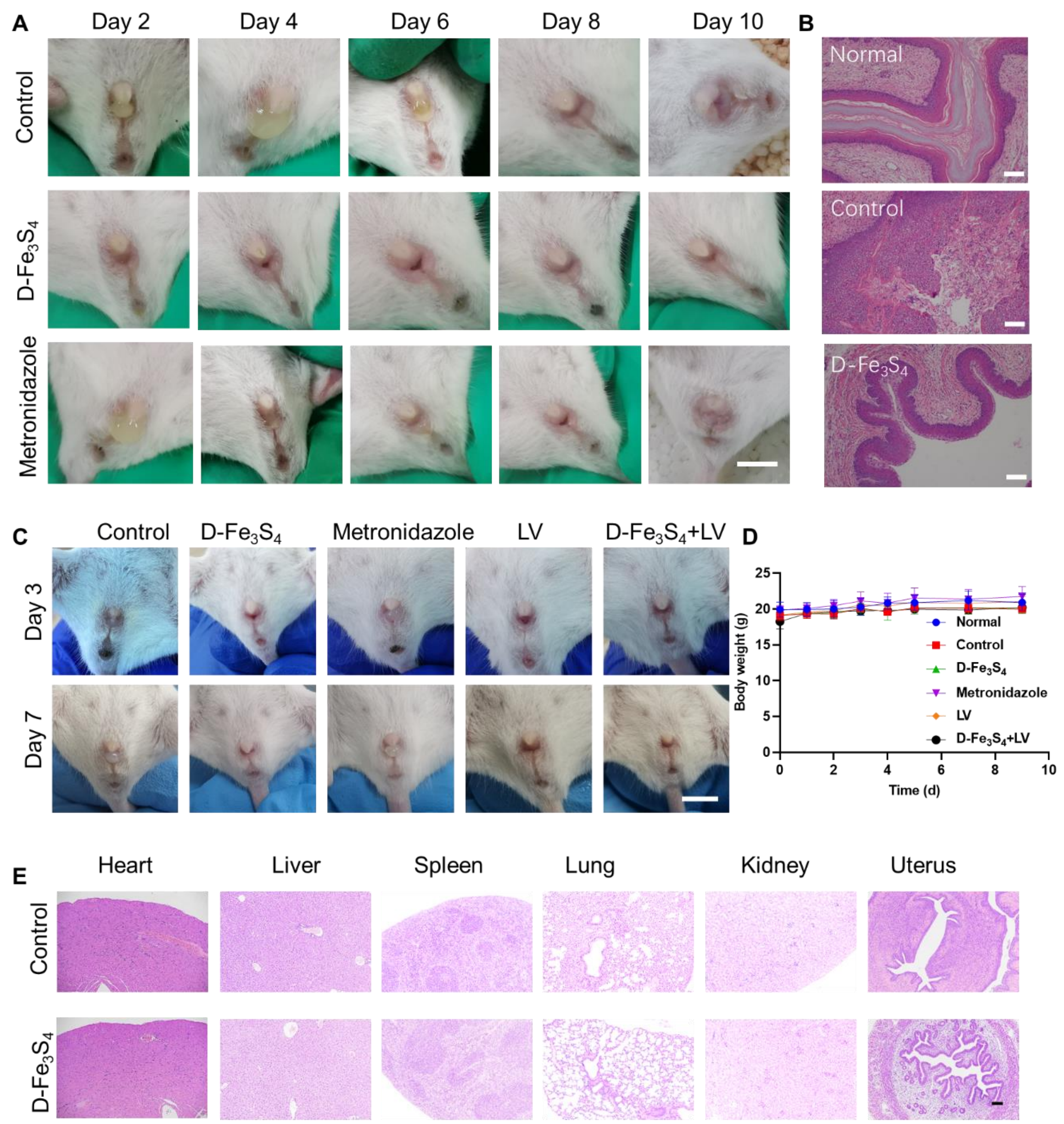

Figure S7. Analyses of D-Fe_3S_4 treating *G. vaginalis* induced vaginosis in mouse models. **A**) Photographs of the mice challenged with bacterial vaginosis treated by D-Fe_3S_4 and metronidazole, respectively. Scale bar: 5 mm. **B**) Histochemistry analysis for vaginal epithelial tissue via D-Fe_3S_4 treatment. The normal group are healthy mice without infection and treatment. Scale bars: 200 µm. **C**) Photographs of *G. vaginalis* infected wounds treated with buffer (control), D-Fe_3S_4, metronidazole, LV, and D-Fe_3S_4 +LV at different times (five mice in each group). Scale bar: 5 mm. **D**) Changes in body weight of mice in different groups. **E**) Histochemistry analysis for organs of mice administered with D-Fe_3S_4. Scale bar: 100 µm. *n* = 5, representative results are shown. Mean±SD are shown.

Table S1 PTS transcription of *G. vaginalis* treated by D-Fe_3S_4.

Description	baseMean_CK	baseMean_T	foldChange(T/CK)
PTS sugar transporter subunit IIC	43.07310756	259.9835211	6.035866365
PTS ascorbate transporter subunit IIC	39329.78014	18155.06876	0.461611245

Note: CK is control group. T is D-Fe_3S_4 group

Table S2. PTS transcription of *G. vaginalis* treated by Na_2S_3.

Description	baseMean_CK	baseMean_M	foldChange(M/CK)
PTS sugar transporter subunit IIC	39.80071419	680.0322212	17.08593012
PTS ascorbate transporter subunit IIC	36347.71363	3555.259631	0.09781247

Note: CK is control group. M is Na_2S_3 group

Table S3 Nugent score for bacterial vaginosis from clinical samples.

Score	*Lactobacillus*（A）	*Gardnerella*/*Bacteroides* (B)	*Mobiluncus*（C）
0	4+（≥30/1000F）	0	0
1	3+（≥30/1000F）	1+	1+ ～2+（＜1～4/1000F）
2	2+（1～4/1000F）	2+	3+ ～4+（5～30/1000F）
3	1+（＜1/1000F）	3+	
4	0（0/1000F）	4+	

Note: Total score is the sum of A+B+C. Evaluation based on total score: 0-3-Normal; 4-6-Intermediate, repeated test later; 7-10-Bacterial vaginosis
